# Citreobenzofuran D–F and Phomenone A–B: Five Novel Sesquiterpenoids from the Mangrove-Derived Fungus *Penicillium* sp. HDN13-494

**DOI:** 10.3390/md20020137

**Published:** 2022-02-13

**Authors:** Qian Wu, Yimin Chang, Qian Che, Dehai Li, Guojian Zhang, Tianjiao Zhu

**Affiliations:** 1 Key Laboratory of Marine Drugs, Chinese Ministry of Education, School of Medicine and Pharmacy, Ocean University of China, Qingdao 266003, China; wqian577@163.com (Q.W.); cheqian064@ouc.edu.cn (Q.C.); dehaili@ouc.edu.cn (D.L.); zhangguojian@ouc.edu.cn (G.Z.); 2 Laboratory for Marine Drugs and Bioproducts, Pilot National Laboratory for Marine Science and Technology (Qingdao), Qingdao 266237, China; yiminchang@163.com

**Keywords:** eremophilane sesquiterpenoids, mangrove-derived fungus, *Penicillium* sp.

## Abstract

Five new sesquiterpenoids, citreobenzofuran D–F (**1**–**3**) and phomenone A–B (**4**–**5**), along with one known compound, xylarenone A (**6**), were isolated from the culture of the mangrove-derived fungus *Penicillium* sp. HDN13-494. Their structures were deduced from extensive spectroscopic data, high-resolution electrospray ionization mass spectrometry (HRESIMS), and electronic circular dichroism (ECD) calculations. Furthermore, the absolute structures of **1** were determined by single-crystal X-ray diffraction analysis. Citreobenzofuran E–F (**2**–**3**) are eremophilane-type sesquiterpenoids with rare benzofuran frameworks, while phomenone A (**4**) contains a rare thiomethyl group, which is the first report of this kind of sesquiterpene with sulfur elements in the skeleton. All the compounds were tested for their antimicrobial and antitumor activity, and phomenone B (**5**) showed moderate activity against *Bacillus subtilis,* with an MIC value of 6.25 μM.

## 1. Introduction

Natural products play an increasingly crucial role in drug discovery, owing to their great chemical and bioactive diversity [1]. As is well known, fungi are a rich source of novel natural products [2], and, in the last two decades, the fungal sources of new metabolites have been broadened from terrestrial strains to marine habitats [3]. The ocean turned out to be an attractive environment, since the search for new biomedicals from marine microorganisms resulted in the isolation of approximately 10,000 metabolites, many of which were endowed with pharmacodynamic properties [4].

Mangrove is a special marine ecosystem that occurs in tropical and subtropical intertidal estuarine zones, and is characterized by high salinity and rich organic matter [5], which makes it an extremely diverse microbial resource. Mangrove-associated fungi, as the second-largest ecological group of marine fungi [6], have proven to be a rich source of natural products, with unique chemical structures and diverse pharmacological activities. Up to now, a large number of metabolites produced by mangrove fungi have been reported, including alkaloids, macrolides, polyketides, quinones, terpenes, and so on [6], which displayed diverse bioactivity, such as antibacterial, insecticidal, antioxidant, and cytotoxic, etc. These findings indicate that mangrove-derived fungi may possess great potential to produce novel and active secondary metabolites. As part of our ongoing work searching for natural products from mangrove-derived fungi, one fungus, *Penicillium* sp. HDN13-494, from the root soil sample of a mangrove plant in Wenchang, Hainan, was selected for further chemical studies. Herein, the details of isolation, structural elucidation, and bioactivities of its metabolites are reported.

## 2. Results and Discussion

**Figure 1:**
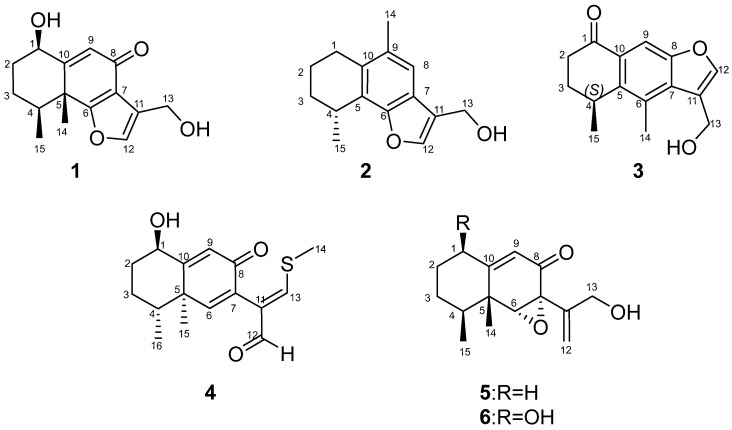
Structures of compounds **1**–**6** isolated from the strain *Penicillium* sp. HDN13-494.

Chemical studies of *Penicillium* sp. HDN13-494 led to the isolation of five new sesquiterpenoids (**1**–**5**) and one known compound (**6**) (Figure 1). Compound **4** represents the first example of eremophilane sesquiterpenoids containing sulfur elements. Citreobenzofuran D (**1**) and E (**2**) possess a rare cyclic system with 6,12-epoxy-eremophilane-type, which has only been reported twice in natural products [7,8].

**Figure 2:**
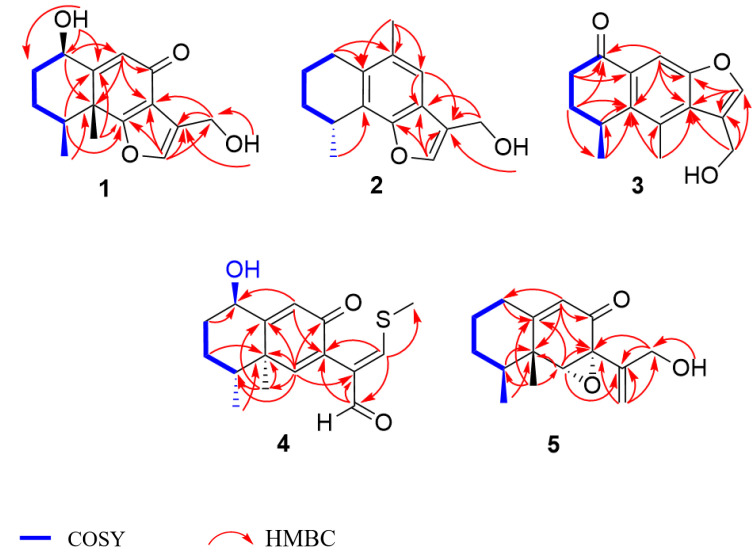
The key HMBC and COSY correlations of **1**–**5**.

**Figure 3:**
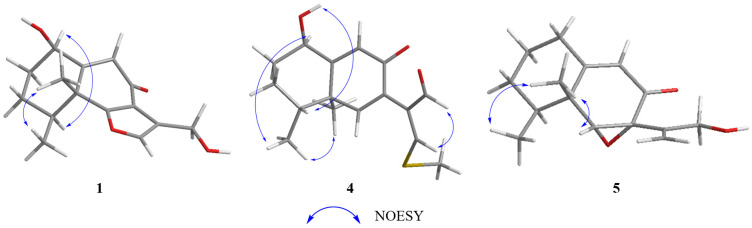
Selected NOESY correlations of compounds **1**, **4** and **5**.

**Figure 4:**
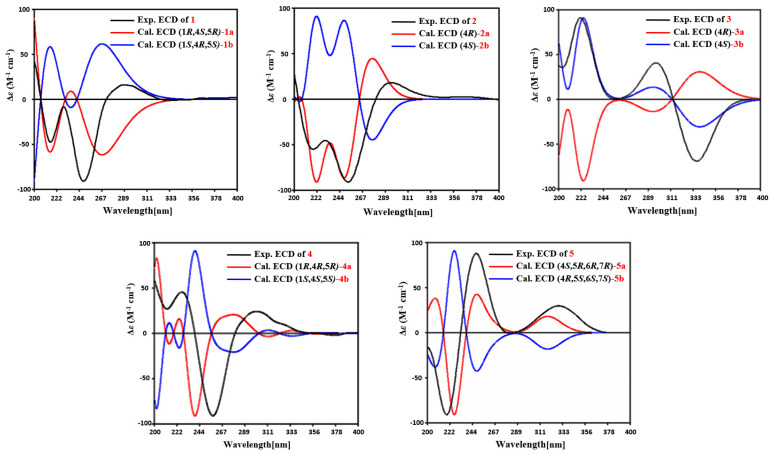
Comparison of calculated ECD spectra and experimental spectra of compounds **1**–**5**.

**Figure 5:**
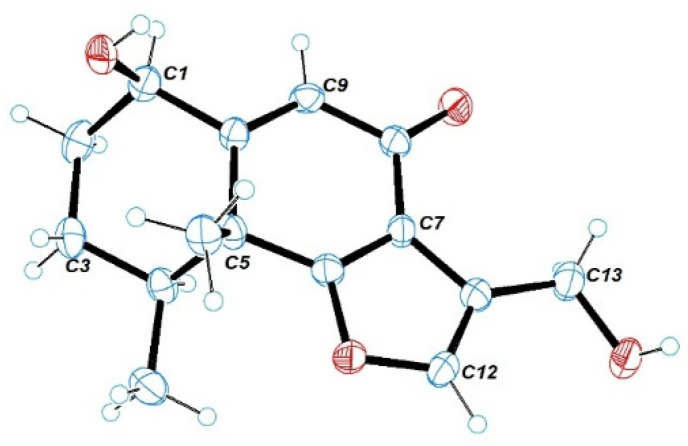
ORTEP drawing for the crystal structure of **1** (35% probability ellipsoids).

Citreobenzofuran D (**1**) was obtained as a brown triclinic crystal with the molecular formula C_15_H_18_O_4_, determined by HRESIMS data (*m*/*z* 261.1130 [M − H]^−^) (Figure S8). The 1D NMR data (Table 1 and Table 2) indicated that **1** shared the same skeleton with isoligularonic acid [7], except for the appearance of two hydroxyl groups at C-1 (*δ*_C_ 72.2) and C-13 (*δ*_C_ 55.2) (Table 1), which was supported by HMBC correlation from OH-1 to C-1, C-2, C-10, and from OH-13 to C-11 and C-13 (Figure 2). In addition, the deshielded singlet at C-9 (*δ*_C_ 125.5) and C-10 (*δ*_C_ 163.5) indicated the occurrence of alkenylation between them (Table 1). The relative configuration of **1** was elucidated as 1R*, 4S*, 5R* on the basis of NOESY correlations of H-1 with H-4, and H-14 with H-15 (Figure 3). To determine the absolute configuration of **1**, the ECD calculations of the optimized conformations of (1*R*, 4*S*, 5*R*)-**1a** and (1*S*, 4*R*, 5*S*)-**1b** were obtained at the B3LYP/6-31+G(d) level. The overall pattern of the experimental ECD spectrum was in reasonable agreement with the calculated ECD spectrum of **1a** (Figure 4), which indicated the 1*R*, 4*S*, and 5*R* absolute configuration of **1**. Single-crystal X-ray diffraction analysis by Cu K*a* radiation further confirmed the absolute configuration of **1** (Figure 5).

Citreobenzofuran E (**2**) was obtained as a green amorphous powder, with the molecular formula C_15_H_18_O_2_, based on the HRESIMS spectrum (*m*/*z* 229.1227 [M − H]^−^) (Figure S15), indicating seven degrees of unsaturation. Detailed analysis of the 1D NMR data of **2** (Table 1 and Table 2) revealed highly structural similarities to the known compound citreobenzofuran B [8]. The difference was the disappearance of the hydroxyl group at C-3 (*δ*_C_ 29.6), supported by the shielded singlet at *δ*_C_ 29.6 (Table 1), and the HSQC correlation between H_2_-3 and C-3 (Figure S12). The absolute configuration of **2** was assigned as 4*R* by comparing the experimental ECD with the calculated ECD (Figure 4).

Citreobenzofuran F (**3**) was obtained as a colorless oil, with the molecular formula C_15_H_16_O_3_, determined by the HRESIMS data (*m*/*z* 243.1032 [M − H]^−^) (Figure S22). The 1D NMR data (Table 1 and Table 2) revealed partial structural similarity to the known compound 3-formyl-4,5-dimethyl-8-oxo-5H-6,7-dihydronaphtho[2,3-*b*] furan [9]. The difference was the replacement of carbonyl at C-13 with a hydroxyl group, which was supported by the chemical shift of C-13 (*δ*_C_ 54.9) (Table 1), and the HSQC correlation between H_2_-13 and C-13 (Figure S19). The absolute configuration of **3** was assigned as 4*S* by comparing the experimental ECD with the calculated ECD (Figure 4).

Phomenone A (**4**) was obtained as a colorless powder. The HRESIMS spectrum suggested that the molecular formula is C_16_H_20_O_3_S (*m*/*z* 293.1205 [M + H]^+^) (Figure S30). The ^1^H and ^13^C NMR spectra of **4** were similar to those of paraconiothin G [10], except for the oxidation of the hydroxyl group at C-12 (*δ*_C_ 188.4) to an aldehyde group, and the appearance of the methylthio group at C-13 (*δ*_C_ 158.0) (Table 1). These changes were confirmed by the HMBC correlations from H-13 (*δ*_H_ 8.01) to C-14 (*δ*_C_ 17.5) and C-12(*δ*_C_ 188.4), and from H-12 (*δ*_H_ 9.28) to C-7(*δ*_C_ 130.8) and C-11(*δ*_C_ 134.9) (Figure 2). The relative configuration of **4** was assigned, by NOESY correlations to between 1-OH and H-4, H-1 and H-15, H-15 and H-16, and H-12 and H-13 (Figure 3). The agreement between the calculated ECD spectrum of (1*R*, 4*R*, 5*R*)*-***4a** and the experimental ECD spectrum suggested that the absolute configuration of **4** is 1*R*, 4*R*, 5*R* (Figure 4).

Phomenone B (**5**) was obtained as a colorless oil, with the molecular formula C_15_H_20_O_3_, determined by HRESIMS analysis (*m*/*z* 247.1343 [M − H]^−^) (Figure S38). According to the 1D NMR data, compound **5** (Table 1 and Table 2) was similar to a known mycotoxic sesquiterpenoid, xylarenones A [11], except for the disappearance of the hydroxyl group at C-1, which was supported by the shielded singlets at *δ*_C_ 32.6 (Table 1). The H-6, 14-CH_3_, and 15-CH_3_ were suggested to be located on the same face of the octahydronaphthalene ring, based on the NOESY correlation of H-6 (*δ*_H_ 3.36) with H-15 (*δ*_H_ 1.03), and H-14 (*δ*_H_ 1.16) with H-15 (Figure 3). Thus, the relative configuration was determined as 4S*, 5R*, 6R*, 7R*. The overall pattern of the experimental ECD spectrum was in reasonable agreement with the calculated ECD spectrum of (4*S*, 5*R*, 6*R*, 7*R*)-**5a** (Figure 4), which indicated the 4*S*, 5*R*, 6*R* and 7*R* absolute configuration of **5**.

The known compound was identified as xylarenones A (**6**) [11] by comparison of the spectroscopic data with the literature values.

Compounds **1**–**6** were tested for their antimicrobial (Table 3) and antitumor activity against K562, MDA-MB-231, L-02, H69AR, and ASPC-1. Compound **5** showed promising antimicrobial activity against *B.* *subtilis,* with an MIC value of 6.25 μM, while the other compounds exerted no activity for all the tested strains. Moreover, none of the compounds displayed obvious anticancer activity.

## 3. Materials and Methods

### 3.1. General Experimental Procedures

Specific rotations were obtained on a JASCO P-1020 digital polarimeter. UV spectra were recorded on a HITACHI 5430. IR spectra were measured on a Bruker Tensor-27 spectrophotometer in KBr discs. NMR spectra were recorded on a JEOLJN M-ECP 600 spectrometer (JEOL, Tokyo, Japan) or an Agilent 500 MHz DD2 spectrometer using tetramethylsilane as an internal standard. HRMS were obtained on a Thermo Scientific LTQ Orbitrap XL mass spectrometer (Thermo Fisher Scientific, Waltham, MA, USA) or Micromass Q-TOF ULTIMA GLOBAL GAA076 LC mass spectrometer (Waters Corporation, Milford, MA, USA). Semipreparative HPLC was performed on an ODS column (YMC-Pack ODS-A, 10 × 250 mm, 5 μm, 3 mL/min).

### 3.2. Materials and Culture Conditions

The fungal strain, *Penicillium* sp. HDN13-494, was isolated from the root soil sample of a mangrove plant in Wenchang. The strain was identified by internal transcribed spacer (ITS) sequence (GenBank No. OM283301). The strain was incubated in media potato dextrose agar (PDA, 20% potato, 2% dextrose, and 2% agar) at 28 °C for 5 days for sporulation. For compound production, the strains were cultured in PDB (potato dextrose broth) at 28 °C, 180 rpm, for 9 days. The strain was deposited at the Marine Medicinal Bioresources Center, Ocean University of China, Qingdao, China.

### 3.3. Fermentation

The strain was grown on PDA plates for 5 days at 28 °C. The spores of *Penicillium* sp. HDN13-494 were inoculated into 500 mL Erlenmeyer flasks containing 150 mL of PDB medium, pH = 7.0 (in seawater collected from Huiquan Bay, Yellow Sea), and cultured at 28 °C for 9 days on a rotary shaker at 180 rpm. A total of 10 L of broth was extracted with EtOAc (4 × 10 L) to generate the extract (30 g).

### 3.4. Extraction and Purification

All the fermentation broth (10 L) was filtered through cheese cloth to separate the supernatant from the mycelia. The supernatant was extracted with EtOAc (4 × 10 L), and the mycelia was macerated and extracted with methanol (3 × 5 L). All extracts were evaporated under reduced pressure to give a crude gum. The extract was chromatographed over ODS, eluting with a gradient of increasing MeOH/H_2_O (30–100%) to afford twelve fractions (Fr.1 to Fr.12). Fr.4 was further separated by RP-HPLC, first using 72% MeOH/H_2_O to yield **4** (3 mg). By using 60% MeCN/H_2_O separated on an RP-HPLC column, compounds **2** (1.4 mg) and **5** (1.4 mg) were obtained from Fr.5, compound **3** (26.4 mg) was obtained from Fr.6 using 55% MeOH/H_2_O, while **1** (1.4 mg) and **6** (2.3 mg) were yielded using 58% MeOH/H_2_O from Fr.7.

Citreobenzofuran D (**1**): brown triclinic crystal (MeOH); m.p. 204–205 °C; ${\left[\alpha \right]}_{D}^{25}$ −42.2 (*c* 0.1, MeOH); UV (MeOH) *λ*_max_ (log *ε*): 231(1.7), 295(0.6) nm; ECD (*c* 0.1 mM, MeOH) *λ*_max_ (Δ *ε*) 216 (−1.00), 249 (−1.90), 288 (0.34) nm; IR (KBr) ν_max_ 3259, 2935, 1655, 1208, 1030 cm^−1^; ^1^H and ^13^C NMR data see Table 1 and Table 2; HRESIMS *m*/*z* 261.1130 [M − H]^−^ (calcd. for C_15_H_17_O_4_, 261.1132).

Citreobenzofuran E (**2**): green amorphous powder; ${\left[\alpha \right]}_{D}^{25}$ −14.1 (*c* 0.1, MeOH); UV (MeOH) *λ*_max_ (log *ε*): 214 (1.6), 279 (0.2) nm; ECD (*c* 0.1 mM, MeOH) *λ*_max_ (Δ *ε*) 216 (−0.53), 252 (−0.78), 292 (0.15) nm; IR (KBr) ν_max_ 3421, 2928, 1684, 1210 cm^−1^; ^1^H and ^13^C NMR data see Table 1 and Table 2; HRESIMS *m*/*z* 229.1227 [M − H]^−^ (calcd. for C_15_H_17_O_2_, 229.1234).

Citreobenzofuran F (**3**): colorless oil; ${\left[\alpha \right]}_{D}^{25}$ −24.0 (*c* 0.1, MeOH); UV (MeOH) *λ*_max_ (log *ε*): 223(1.5), 295(1.0) nm; ECD (*c* 0.1 mM, MeOH) *λ*_max_ (Δ *ε*) 211 (0.47), 298 (0.13), 338 (6.91) nm; IR (KBr) ν_max_ 3409, 2927, 1668, 1675, 1176 cm^−1^; ^1^H and ^13^C NMR data see Table 1 and Table 2; HRESIMS *m*/*z* 243.1032 [M − H]− (calcd. for C_15_H_15_O_3_, 243.1027).

Phomenone A (**4**): colorless powder; ${\left[\alpha \right]}_{D}^{25}$ −4.3 (*c* 0.1, MeOH); UV (MeOH) *λ*_max_ (log *ε*): 201(1.6), 221(1.5), 298 (1.3) nm; ECD (*c* 0.1 mM, MeOH) *λ*_max_ (Δ *ε*) 211 (0.47), 298 (0.13), 338 (6.91) nm; IR (KBr) ν_max_ 3420, 2930, 1675, 1208 cm^−1^; ^1^H and ^13^C NMR data see Table 1 and Table 2; HRESIMS *m*/*z* 293.1205 [M + H]^+^ (calcd. for C_16_H_21_O_3_S, 293.1206).

Phomenone B (**5**): colorless oil; ${\left[\alpha \right]}_{D}^{25}$ +336.24 (*c* 0.1, MeOH); UV (MeOH) *λ*_max_ (log *ε*): 202(0.5), 248(1.8) nm; ECD (*c* 0.1 mM, MeOH) *λ*_max_ (Δ *ε*) 216 (−6.80), 248 (7.00), 329 (2.37) nm; IR (KBr) ν_max_ 3749, 2935, 1671 cm^−1^; ^1^H and ^13^C NMR data see Table 1 and Table 2; HRESIMS *m*/*z* 247.1343 [M − H]^−^ (calcd. for C_15_H_19_O_3_, 247.1340).

### 3.5. X-ray Crystal Structure Analysis

Compound **1** was obtained as brown triclinic crystals from MeOH with the molecular formula C_31_H_40_O_9_. The suitable crystal was selected and analyzed on a CCD area detector diffractometer (Bruker Smart Apex II) using Cu K*a* radiation. The crystallographic data for **1** (CCDC 2128584) was deposited in the Cambridge Crystallographic Data Centre.

Crystal data for citreobenzofuran D (**1**): C_31_H_40_O_9_ (*M* = 556.63), triclinic, space group P1, *a* = 7.6499(4) Å, *b* = 9.7072(7) Å, *c* = 11.2321(7) Å, α = 103.630(6)°, *β* = 90.229(5)°, γ = 104.053(5)°, *V* = 721.01(8) Å^3^, *Z* = 1, *T* = 293(2) K, *μ* (Cu K*α*) = 0.770 mm^−1^, D_calc_ = 1.282 g/cm^3^, 4156 reflections measured (4.417° ≤ θ ≤ 67.244°), 2898 unique (*R*_int_ = 0.0232), which were used in all calculations. The final *R*_1_ was 0.0351 (*I* > 2*σ*(I)) and ω*R*_2_ was 0.0945, flack = −0.1(2).

### 3.6. Computational Section

Conformational searches were performed by employing the systematic procedure implemented in *Spartan*′14, using the MMFF (Merck molecular force field). All MMFF minima were reoptimized with DFT calculations at the B3LYP/6-31+G(d) level using the Gaussian09 program. The geometry was optimized starting from various initial conformations, with vibrational frequency calculations confirming the presence of minima. Time-dependent DFT calculations were performed on lowest-energy conformations (>5% population) for each configuration using 20 excited states and using a polarizable continuum model for MeOH. ECD spectra were generated using the program SpecDis by applying a Gaussian band shape with a 0.30 eV width and 25 blue shifts to facilitate comparison to the experimental data.

### 3.7. Cytotoxicity Assay

Cytotoxic activities of compounds **1**–**6** were evaluated against the K562 (using the MTT method), MDA-MB-231, L-02, H69AR and ASPC-1 (using the SRB method) cell lines. Adriamycin (ADM) was used as a positive control. The detailed methodologies for biological testing have been described in previous reports [12,13].

### 3.8. Antimicrobial Activity

Antibacterial activities of compounds **1**–**6** were evaluated against *B. subtilis, Proteus vulgaris, Acinetobacter baumannii, Candida. albicans, Escherichia. coli,* and MRSA (Methicillin-resistant *Staphylococcus aureus*) by using the agar dilution method as previously reported [14]. Ciprofloxacin was used as a positive control.

## 4. Conclusions

In summary, five new sesquiterpenoids, citreobenzofuran D–F (**1**–**3**) and phomenone A–B (**4**–**5**), along with one known compound, xylarenones A (**6**), were isolated from a mangrove-derived fungus, *Penicillium* sp. HDN13-494. Compounds **1**, **4** and **5** are canonical eremophilane structures, while compounds **2** and **3** possess rare benzofuran structures in the skeletons. The biological activity assay showed that compound **5** possessed mild antimicrobial activity against *Bacillus subtilis*. Our research results expanded the diversity of the secondary metabolite in mangrove-derived fungi, and also enriched the diversity of terpenoids in natural products.

## Figures and Tables

**Table 1:** ^13^C NMR data for compounds **1**–**5** in DMSO-*d*_6_.

No.	1 ^a^	2 ^b^	3 ^a^	4 ^a^	5 ^b^
1	72.2	27.0	197.9	72.3	32.6
2	34.9	18.7	33.1	34.9	26.8
3	25.8	29.6	29.1	25.4	30.0
4	42.8	27.5	28.6	42.1	38.1
5	44.6	125.7	141.9	44.1	41.1
6	172.8	152.8	129.7	158.0	69.2
7	117.2	124.2	131.7	130.8	62.0
8	183.8	118.4	153.9	183.3	120.3
9	125.5	131.4	107.6	125.5	131.4
10	163.5	130.8	129.0	167.2	166.7
11	124.5	121.7	122.7	134.9	145.6
12	140.4	141.5	147.5	188.4	111.6
13	55.2	54.5	54.9	158.0	62.2
14	18.4	20.2	14.8	17.5	17.3
15	17.8	21.52	19.4	19.0	16.0
16	--	--	--	16.4	--

^a^ Spectra were recorded at 125 MHz for ^13^C NMR using DMSO-*d*_6_ as solvent. ^b^ Spectra were recorded at 100 MHz for ^13^C NMR using DMSO-*d*_6_ as solvent.

**Table 2:** ^1^H NMR data for compounds **1**–**5** in DMSO-*d*_6_.

No.	1 ^c^	2 ^c^	3 ^d^	4 ^c^	5 ^c^
1	4.52 (q, 2.9)	2.52 (m), ovp. 2.67 (dd,17.2, 4.4)	--	5.22 (m)	2.40 (tdd, 13.7, 5.3, 2.0) 2.67 (dd,17.2, 4.4)
2	1.88 (m), ovp. 1.51 (m), ovp.	1.81 (m), ovp.	2.86 (m) 2.46 (m), ovp.	1.88 (m), ovp. 1.53 (m), ovp.	1.30 (m) 1.77 (m), ovp.
3	1.84 (m), ovp. 1.44 (m), ovp.	1.67 (s) 1.80 (m), ovp.	2.19 (m) 2.02 (d, 11.2)	1.81 (m), ovp. 1.38 (m), ovp.	1.48 (m)
4	1.54 (m), ovp.	3.31 (m), ovp.	3.46 (s)	1. 47 (m), ovp.	1.73 (m), ovp.
5	--	--	--	--	--
6	--	--	--	6.93 (s)	3.36 (s)
7	--	--	--	--	--
8	--	7.26 (s)	--	--	5.67 (d, 1.9)
9	6.13 (s)	--	7.85 (s)	6.13 (s)	--
10	--	--	--	--	--
11	--	--	--	--	--
12	7.63 (s)	7.71 (s)	8.02 (s)	9.28 (s)	5.18 (q, 1.8) 5.06 (q, 1.5)
13	4.57 (dd, 5.5, 1.4)	4.56 (d, 4.6)	4.70 (s)	8.01 (s)	4.05 (m)
14	1.51 (s)	2.23 (s)	2.64 (s)	2.50 (m). ovp.	1.16 (s)
15	1.22 (d, 6.7)	1.31 (d, 7.0)	1.27 (d, 7.0)	1.30 (s)	1.03 (d, 6.8)
16	--	--	--	1.03 (d, 6.7)	--
1-OH	5.33 (d, 2.7)	--	--	4.37 (d, 2.9)	--
13-OH	4.99 (t, 5.6)	5.06 (br t, 5.4)	--	--	4.84 (dd, 6.6, 4.5)

^c^ Spectra were recorded at 500 MHz for ^1^H NMR using DMSO-*d*_6_ as solvent. ^d^ Spectra were recorded at 400 MHz for ^1^H NMR using DMSO-*d*_6_ as solvent.

**Table 3:** MIC values (μM) of compounds **1**–**6**.

Compounds	MIC(μM)					
	*Bacillus. subtilis*	*Proteus vulgaris*	*Acinetobacter baumannii*	*Candida. albicans*	*Escherichia. coli*	MRSA
Citreobenzofuran D (**1**)	>50	>50	>50	>50	>50	>50
Citreobenzofuran E (**2**)	>50	>50	>50	>50	>50	>50
Citreobenzofuran F (**3**)	>50	>50	>50	>50	>50	>50
Phomenone A (**4**)	>50	>50	>50	>50	50	>50
Phomenone B (**5**)	6.25	>50	>50	>50	>50	>50
xylarenones A (**6**)	>50	>50	>50	>50	>50	>50

## Data Availability

Data are contained within the article.

## Supplementary Materials

The following are available online at https://www.mdpi.com/article/10.3390/md20020137/s1: Figure S1: HPLC analysis of the crude extract of *Penicillium* sp. HDN13-494; Figures S2–S9: 1D, 2D NMR, HRESIMS and IR spectra of compound **1**; Figures S10–S16: 1D, 2D NMR, HRESIMS and IR spectra of compound **2**; Figures S17–S23: 1D, 2D NMR, HRESIMS and IR spectra of compound **3**; Figures S24–S31: 1D, 2D NMR, HRESIMS and IR spectra of compound **4**; Figures S32–S39: 1D, 2D NMR, HRESIMS and IR spectra of compound **5**; Figures S40–S44: DFT-optimized structures for low-energy conformers of **1**–**5** at B3LYP/TZVP level in DMSO (CPCM) (conformer populations were calculated using the Gibbs free energy and Boltzmann population at 298 K estimated thereof); Tables S1–S5: harmonic frequencies of **1**–**5**; Tables S6–S10: important thermodynamic parameters (a.u.) and Boltzmann distributions of the optimized compounds **1**–**5** at B3LYP/TZVP level of theory with PCM solvent model for DMSO.

## References

[1] Casertano M., Menna M., Imperatore C. (2020). The Ascidian-Derived Metabolites with Antimicrobial Properties. Antibiotics.

[2] Carroll A.R., Copp B.R., Davis R.A., Keyzers R.A., Prinsep M.R. (2020). Marine Natural Products. Nat. Prod. Rep..

[3] Schueffler A., Anke T. (2014). Fungal Natural Products in Research and Development. Nat. Prod. Rep..

[4] Kavisri M., Abraham M., Prabakaran G., Elangovan M. (2021). Meivelu Moovendhan Phytochemistry, Bioactive Potential and Chemical Characterization of Metabolites from Marine Microalgae (*Spirulina platensis*) Biomass. Biomass Convers. Biorefin..

[5] Cadamuro R.D., da Silveira Bastos I.M.A., da Silva I.T., da Cruz A.C.C., Robl D., Sandjo L.P., Alves S., Lorenzo J.M., Rodríguez-Lázaro D., Treichel H. (2021). Bioactive Compounds from Mangrove Endophytic Fungus and Their Uses for Microorganism Control. J. Fungi.

[6] Chen S., Cai R., Liu Z., Cui H., She Z. (2022). Secondary Metabolites from Mangrove-Associated Fungi: Source, Chemistry and Bioactivities. Nat. Prod. Rep..

[7] Yuan Y.L., Fang L.Z., Bai S.P., Liang H.J., Ye D.D. (2013). A Novel Eremophilane Sesquiterpenoid from *Ligularia intermedia* Roots and Rhizomes. Chem. Nat. Compd..

[8] Kosemura S. (2003). Meroterpenoids from *Penicillium citreo-viride* B. IFO 4692 and 6200 Hybrid. Tetrahedron.

[9] Vishnoi S.P., Shoeb A., Kapil R.S., Popli S.P. (1983). A Furanoeremophilane from *Vitex negundo*. Phytochemistry.

[10] Nakashima K.I., Tomida J., Hirai T., Kawamura Y., Inoue M. (2019). Paraconiothins A-J: Sesquiterpenoids from the Endophytic Fungus *Paraconiothyrium brasiliense* ECN258. J. Nat. Prod..

[11] Hu Z.Y., Li Y.Y., Huang Y.J., Su W.J., Shen Y.M. (2008). Three New Sesquiterpenoids from *Xylaria* Sp. NCY2. Helv. Chim. Acta.

[12] Mosmann T. (1983). Rapid Colorimetric Assay for Cellular Growth and Survival: Application to Proliferation and Cytotoxicity Assays. J. Immunol. Methods.

[13] Bringmann G., Lang G., Maksimenka K., Hamm A., Gulder T.A.M., Dieter A., Bull A.T., Stach J.E.M., Kocher N., Müller W.E.G. (2005). Gephyromycin, the First Bridged Angucyclinone, from *Streptomyces Griseus* Strain NTK 14. Phytochemistry.

[14] Yu G., Wu G., Sun Z., Zhang X., Che Q., Gu Q., Zhu T., Li D., Zhang G. (2018). Cytotoxic Tetrahydroxanthone Dimers from the Mangrove-Associated Fungus *Aspergillus versicolor* HDN1009. Mar. Drugs.

