# Supplementary material for: Citreobenzofuran D–F and Phomenone A–B: Five Novel Sesquiterpenoids from the Mangrove-Derived Fungus Penicillium sp. HDN13-494
Source: Mar Drugs. 2022 Feb 13;20(2):137. doi: 10.3390/md20020137 (PMC8878823; doi:10.3390/md20020137)
Supplement: Supplementary file 1 [file marinedrugs-20-00137-s001.zip › marinedrugs-1595556-SI.pdf]

Supporting Information

Citreobenzofuran D−F and Phomenone A−B: Five Novel Sesquiterpenoids from the Mangrove Endophytic Fungus *Penicillium* sp. HDN13-494

Qian Wu [1], Yiming Chang [1], Qian Che [1], Guojian Zhang [1,2], Dehai Li [1,2] and Tianjiao Zhu [1,2,*]

[1] Key Laboratory of Marine Drugs, Chinese Ministry of Education, School of Medicine and Pharmacy, Ocean University of China, Qingdao 266003, China

[2] Laboratory for Marine Drugs and Bioproducts, Pilot National Laboratory for Marine Science and Technology (Qingdao), Qingdao 266237, China

[*] Correspondence: zhutj@ouc.edu.cn; Tel.: +86-532-82031632; Fax: +86-532-8203-3054

Table of Contents

Figure S1. HPLC analysis of the crude extract of *Penicillium* sp. HDN13-494. 4
Figure S2. ^{1}H NMR (500 MHz, DMSO) spectrum of compound **1**. 4
Figure S3. ^{13}C NMR (125 MHz, DMSO) spectrum of compound **1**. 4
Figure S4. HSQC spectrum of compound **1**. 5
Figure S5. ^{1}H-^{1}H COSY (500 MHz, DMSO) spectrum of compound **1**. 5
Figure S6. HMBC spectrum of compound **1**. 6
Figure S7. NOESY (400 MHz, DMSO) spectrum of compound **1**. 6
Figure S8. The HRESIMS spectrum of compound **1**. 7
Figure S9. The IR spectrum of compound **1**. 8
Figure S10. ^{1}H NMR (500 MHz, DMSO) spectrum of compound **2**. 8
Figure S11. ^{13}C NMR (100 MHz, DMSO) spectrum of compound **2**. 8
Figure S12. HSQC spectrum of compound **2**. 9
Figure S13. ^{1}H-^{1}H COSY (500 MHz, DMSO) spectrum of compound **2**. 9
Figure S14. HMBC spectrum of compound **2**. 10
Figure S15. The HRESIMS spectrum of compound **2**. 10
Figure S16. The IR spectrum of compound **2**. 11
Figure S17. ^{1}H NMR (400 MHz, DMSO) spectrum of compound **3**. 11
Figure S18. ^{13}C NMR (125 MHz, DMSO) spectrum of compound **3**. 12
Figure S19. HSQC spectrum of compound **3**. 12
Figure S20. ^{1}H-^{1}H COSY (500 MHz, DMSO) spectrum of compound **3**. 13
Figure S21. HMBC spectrum of compound **3**. 13
Figure S22. The HRESIMS spectrum of compound **3**. 14
Figure S23. The IR spectrum of compound **3**. 14
Figure S24. ^{1}H NMR (500 MHz, DMSO) spectrum of compound **4**. 15
Figure S25. ^{13}C NMR (125 MHz, DMSO) spectrum of compound **4**. 15
Figure S26. HSQC spectrum of compound **4**. 16
Figure S27. ^{1}H-^{1}H COSY (500 MHz, DMSO) spectrum of compound **4**. 17
Figure S28. HMBC spectrum of compound **4**. 17
Figure S29. NOESY (500 MHz, DMSO) spectrum of compound **4**. 18
Figure S30. The HRESIMS spectrum of compound **4**. 18
Figure S31. The IR spectrum of compound **4**. 19
Figure S32. ^{1}H NMR (500 MHz, DMSO) spectrum of compound **5**. 19
Figure S33. ^{13}C NMR (125 MHz, DMSO) spectrum of compound **5**. 19
Figure S34. HSQC spectrum of compound **5**. 20
Figure S35. ^{1}H-^{1}H COSY (500 MHz, DMSO) spectrum of compound **5**. 20
Figure S36. HMBC spectrum of compound **5**. 21
Figure S37. NOESY (500 MHz, DMSO) spectrum of compound **5**. 21
Figure S38. The HRESIMS spectrum of compound **5**. 22
Figure S39. The IR spectrum of compound **5**. 22
Figure S40. DFT-optimized structures for low-energy conformers of (1*R*, 4*S*, 5*R*)-**1** at B3LYP/TZVP level in DMSO (CPCM) (Conformer populations were calculated using the Gibbs free energy and Boltzmann population at 298 K estimated thereof). 23
Figure S41. DFT-optimized structures for low-energy conformers of (4*R*)-**2** at B3LYP/TZVP level

in DMSO (CPCM) (Conformer populations were calculated using the Gibbs free energy and Boltzmann population at 298 K estimated thereof). 23
Figure S42. DFT-optimized structures for low-energy conformers of (4*R*)-**3** at B3LYP/TZVP level in DMSO (CPCM) (Conformer populations were calculated using the Gibbs free energy and Boltzmann population at 298 K estimated thereof). 24
Figure S43. DFT-optimized structures for low-energy conformers of (4*R*)-**4** at B3LYP/TZVP level in DMSO (CPCM) (Conformer populations were calculated using the Gibbs free energy and Boltzmann population at 298 K estimated thereof). 25
Figure S44. DFT-optimized structures for low-energy conformers of (4*S*,5*R*,6*R*,7*R*)-**5** at B3LYP/TZVP level in DMSO (CPCM) (Conformer populations were calculated using the Gibbs free energy and Boltzmann population at 298 K estimated thereof). 26
Table S1. Harmonic frequencies (cm**-**1**) of **1**..27
Table S2. Harmonic frequencies (cm**-**2**) of **2**..27
Table S3. Harmonic frequencies (cm**-**3**) of **3**..27
Table S4. Harmonic frequencies (cm**-**4**) of **4**..27
Table S5. Harmonic frequencies (cm**-**5**) of **5**..28
Table S6. Important thermodynamic parameters (a.u.) and Boltzmann distributions of the optimized-**1** at B3LYP/TZVP level of theory with PCM solvent model for DMSO. 28
Table S7. Important thermodynamic parameters (a.u.) and Boltzmann distributions of the optimized-**2** at B3LYP/TZVP level of theory with PCM solvent model for DMSO. 28
Table S8. Important thermodynamic parameters (a.u.) and Boltzmann distributions of the optimized-**3** at B3LYP/TZVP level of theory with PCM solvent model for DMSO. 28
Table S9. Important thermodynamic parameters (a.u.) and Boltzmann distributions of the optimized-**4** at B3LYP/TZVP level of theory with PCM solvent model for DMSO. 28
Table S10. Important thermodynamic parameters (a.u.) and Boltzmann distributions of the optimized-**5** at B3LYP/TZVP level of theory with PCM solvent model for DMSO. 29

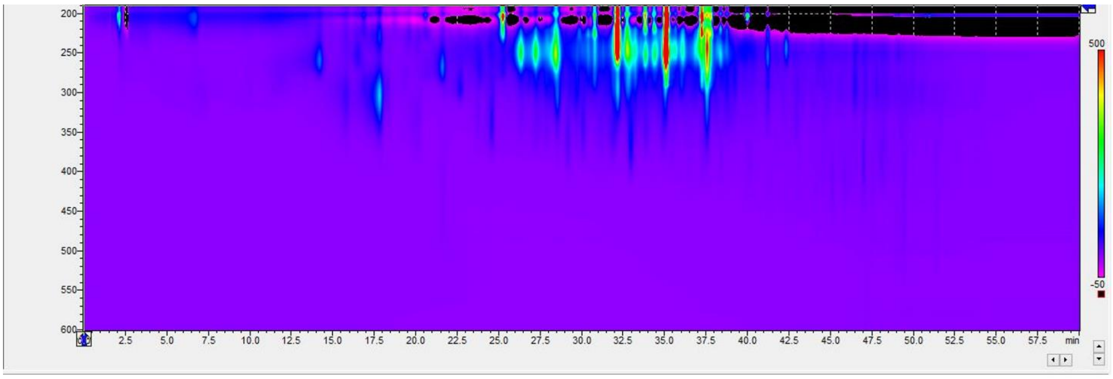

Figure S1. HPLC analysis of the crude extract of *Penicillium* sp. HDN13-494.

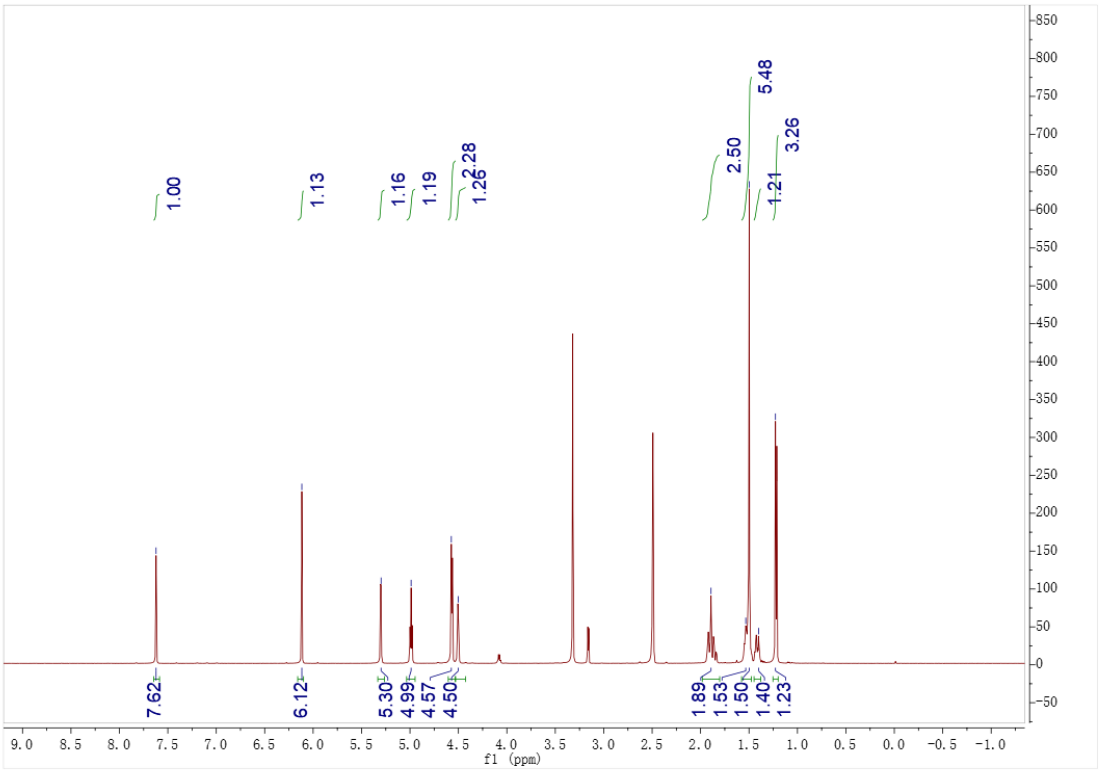

Figure S2. ^{1}H NMR (500 MHz, DMSO) spectrum of compound **1**.

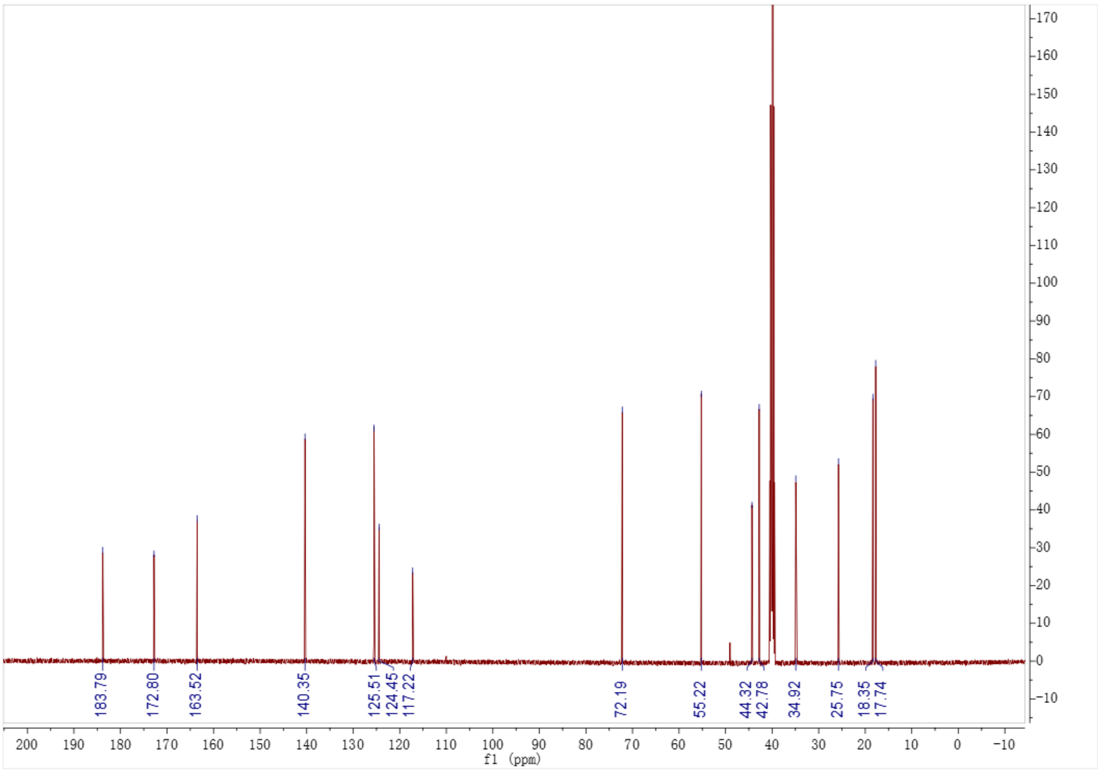

Figure S3. ^{13}C NMR (125 MHz, DMSO) spectrum of compound **1**.

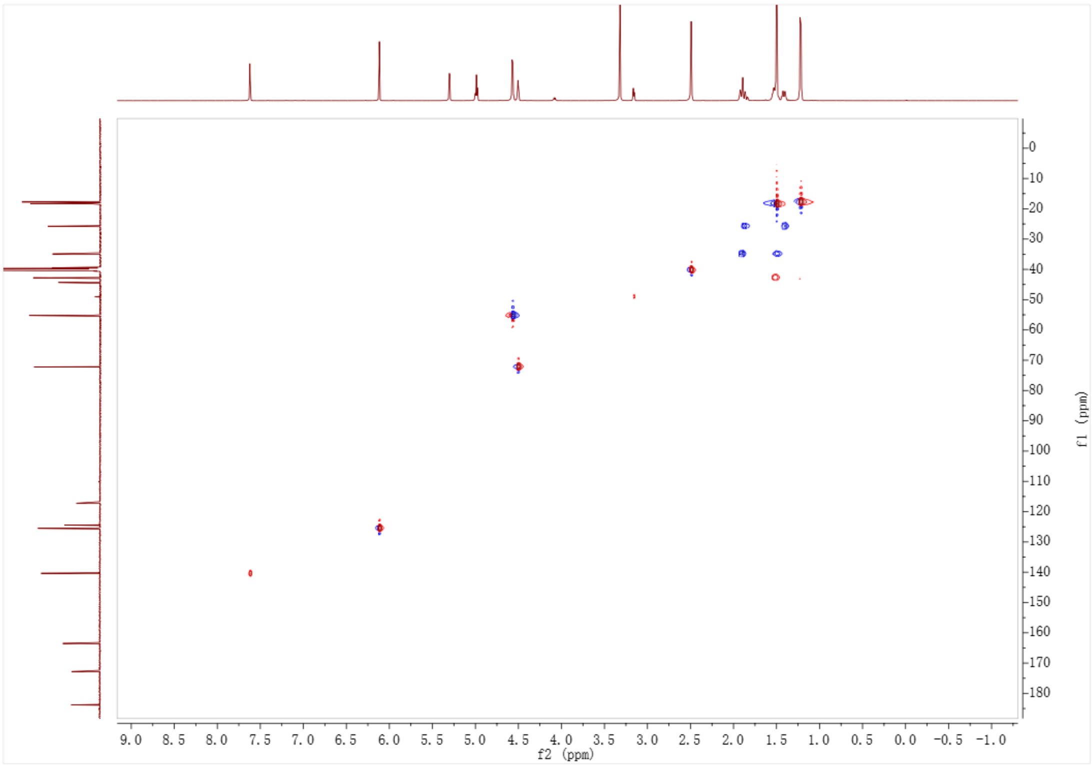

Figure S4. HSQC spectrum of compound **1**.

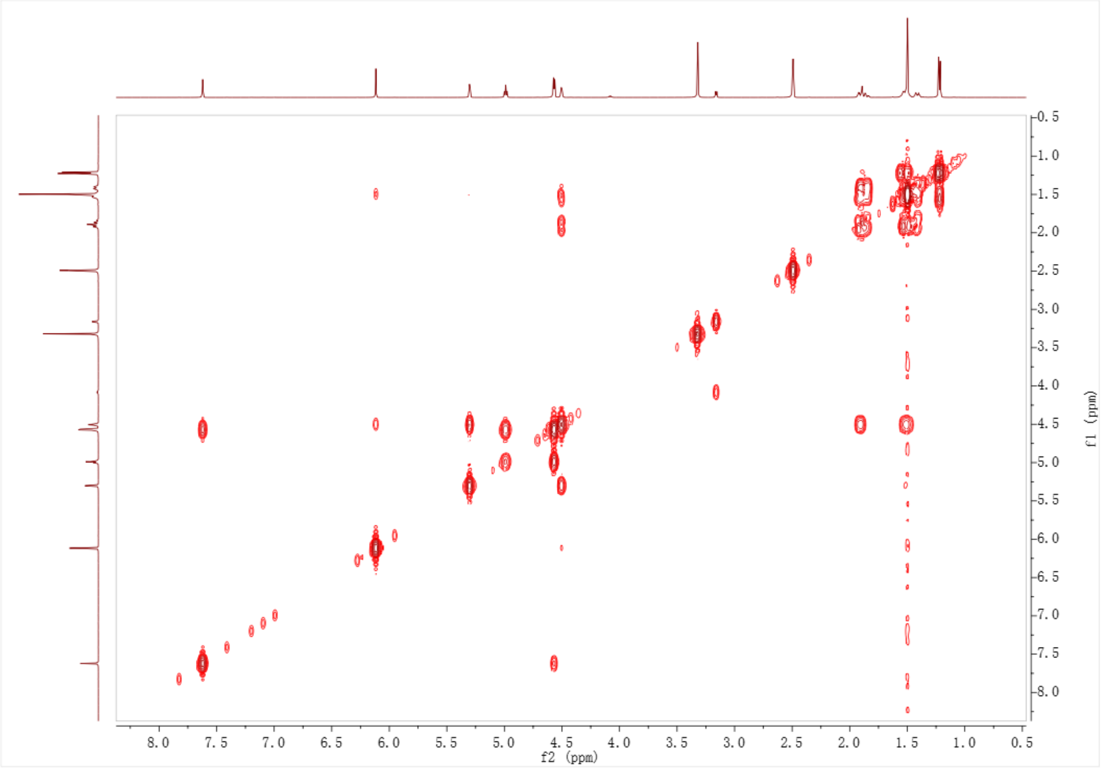

Figure S5. ^{1}H-^{1}H COSY (500 MHz, DMSO) spectrum of compound **1**.

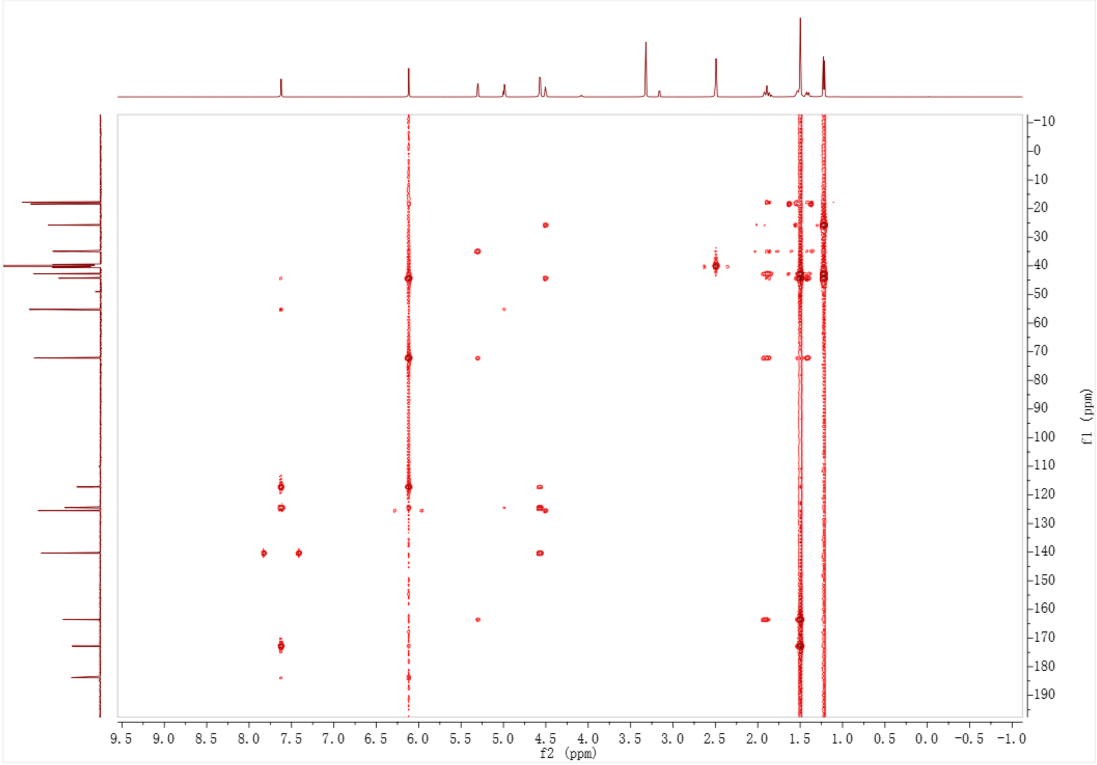

Figure S6. HMBC spectrum of compound **1**.

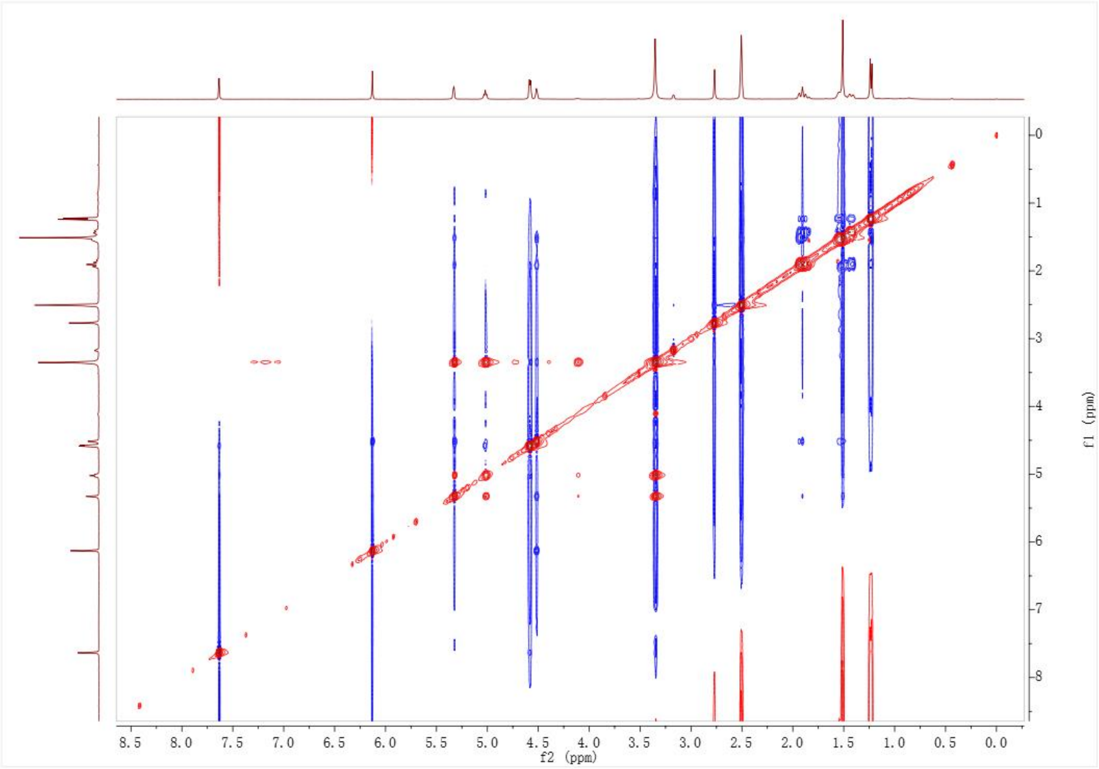

Figure S7. NOESY (400 MHz, DMSO) spectrum of compound **1**.

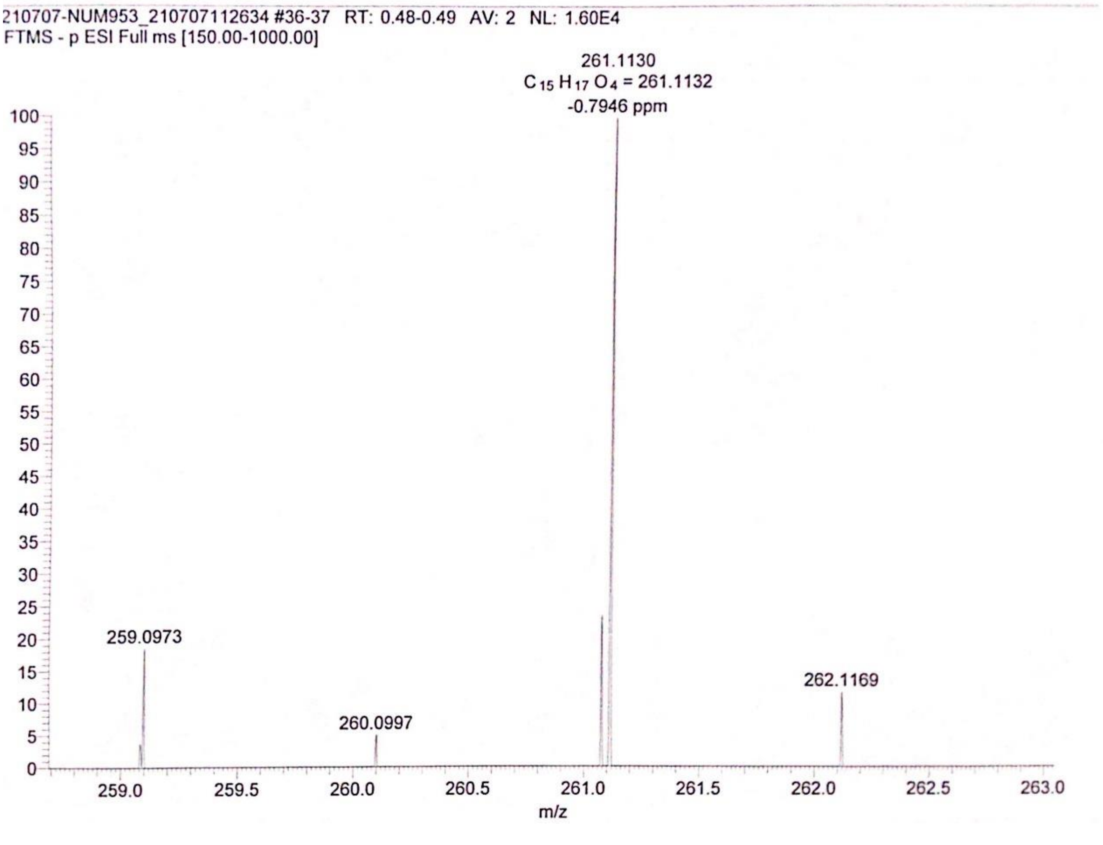

Figure S8. The HRESIMS spectrum of compound **1**.

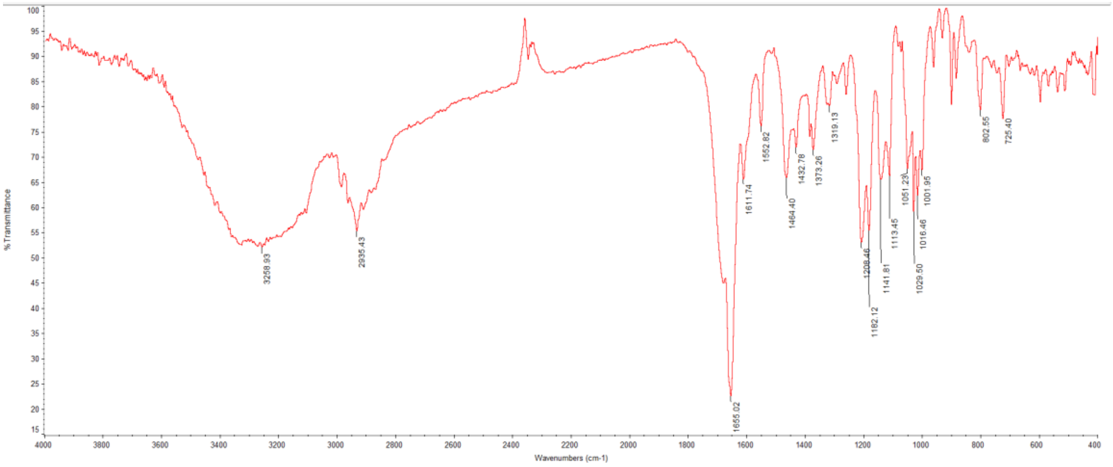

Figure S9. The IR spectrum of compound **1**.

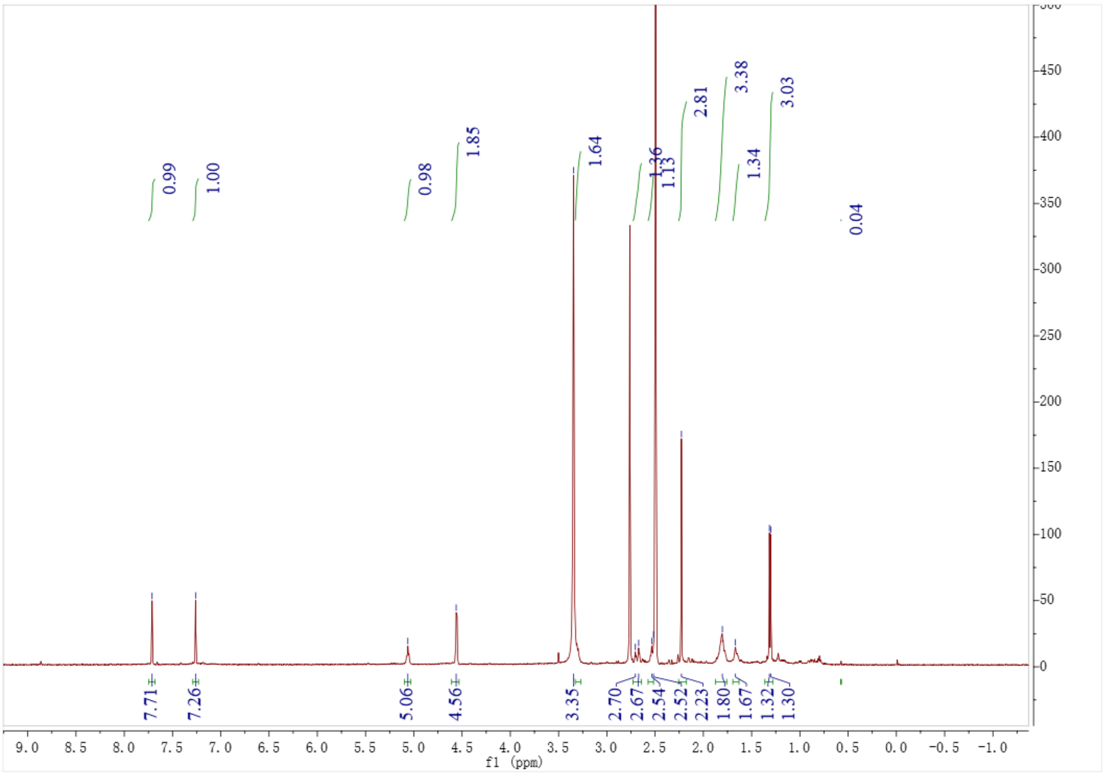

Figure S10. ^{1}H NMR (500 MHz, DMSO) spectrum of compound **2**.

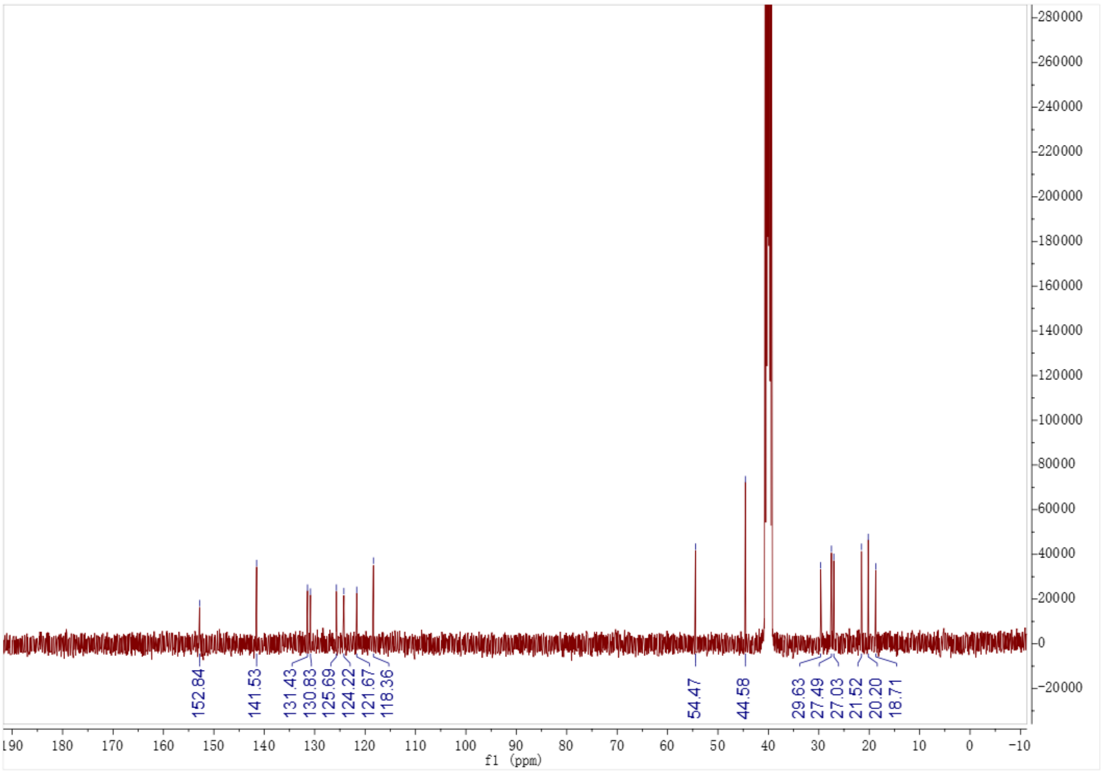

Figure S11. ^{13}C NMR (100 MHz, DMSO) spectrum of compound **2**.

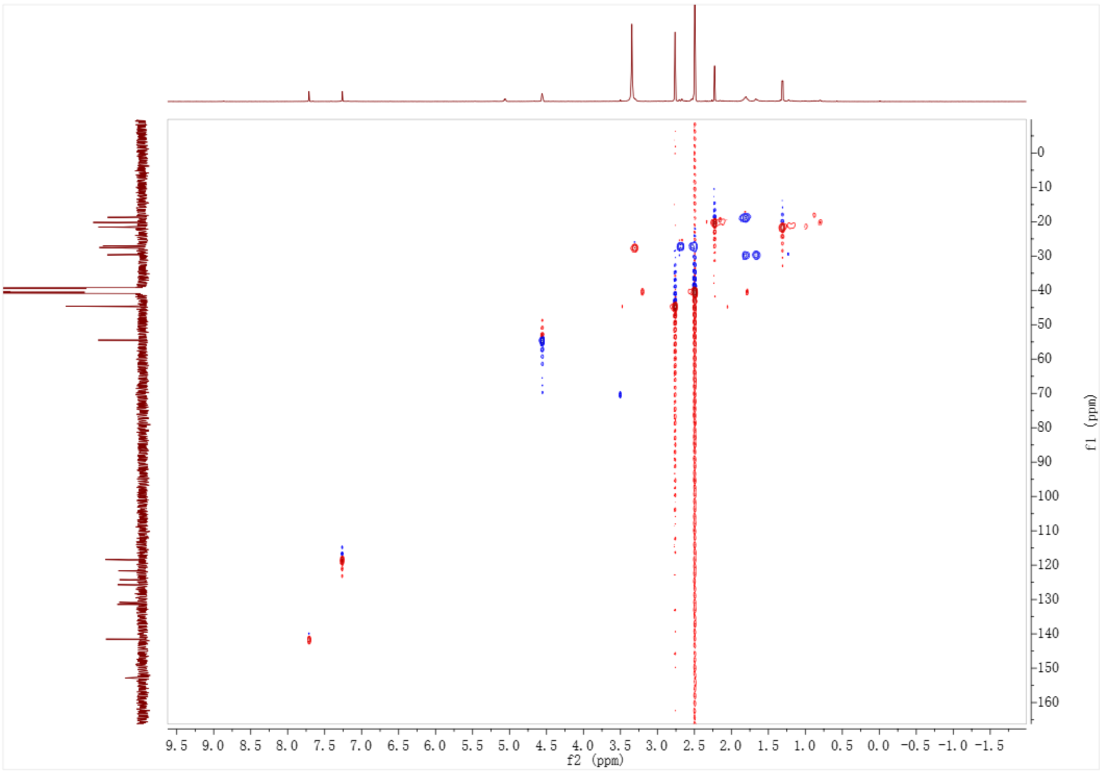

Figure S12. HSQC spectrum of compound **2**.

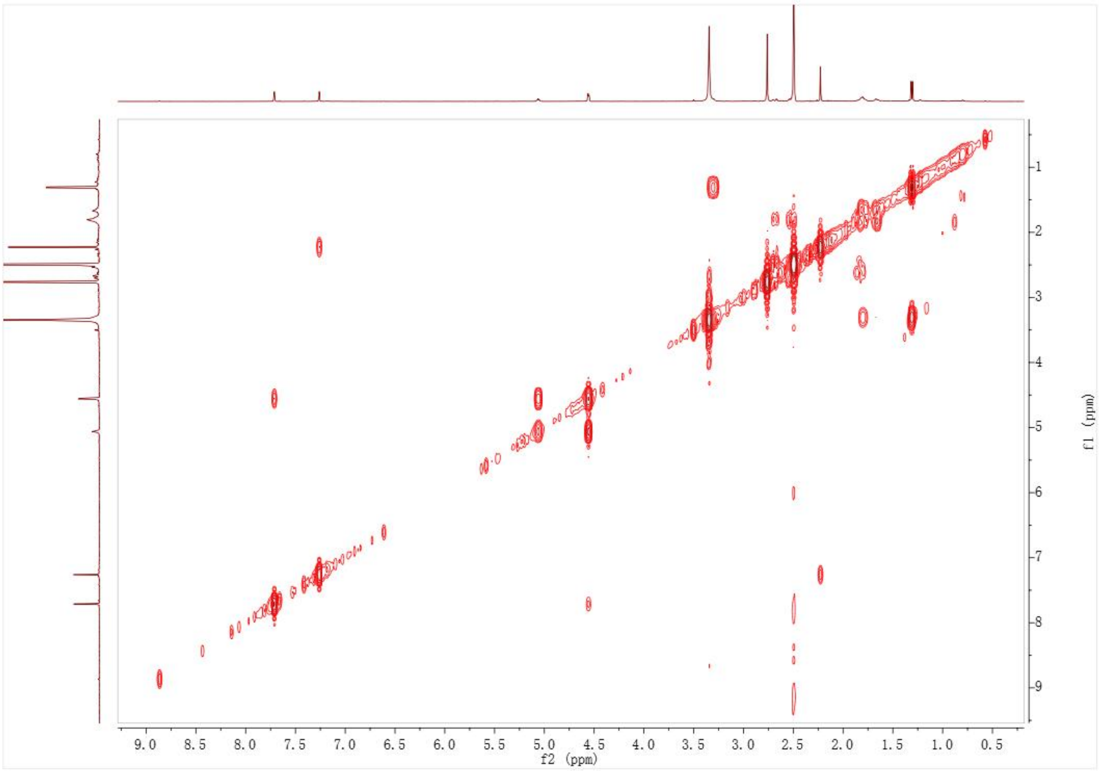

Figure S13. ^{1}H-^{1}H COSY (500 MHz, DMSO) spectrum of compound **2**.

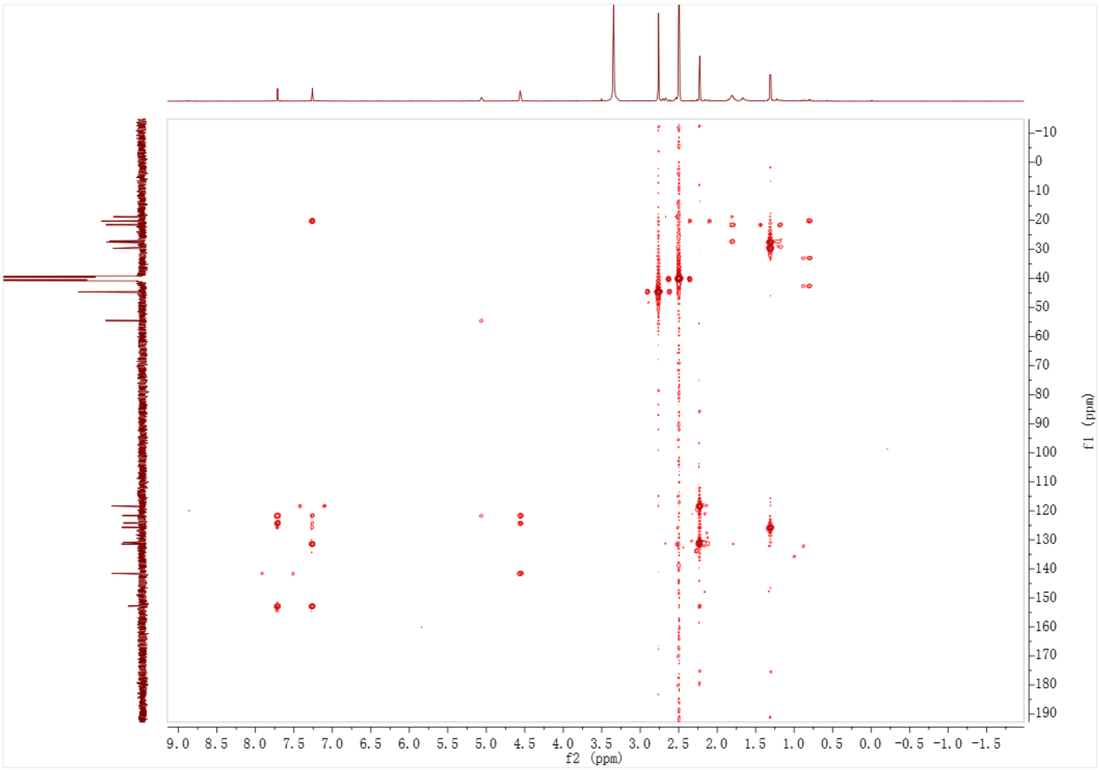

Figure S14. HMBC spectrum of compound **2**.

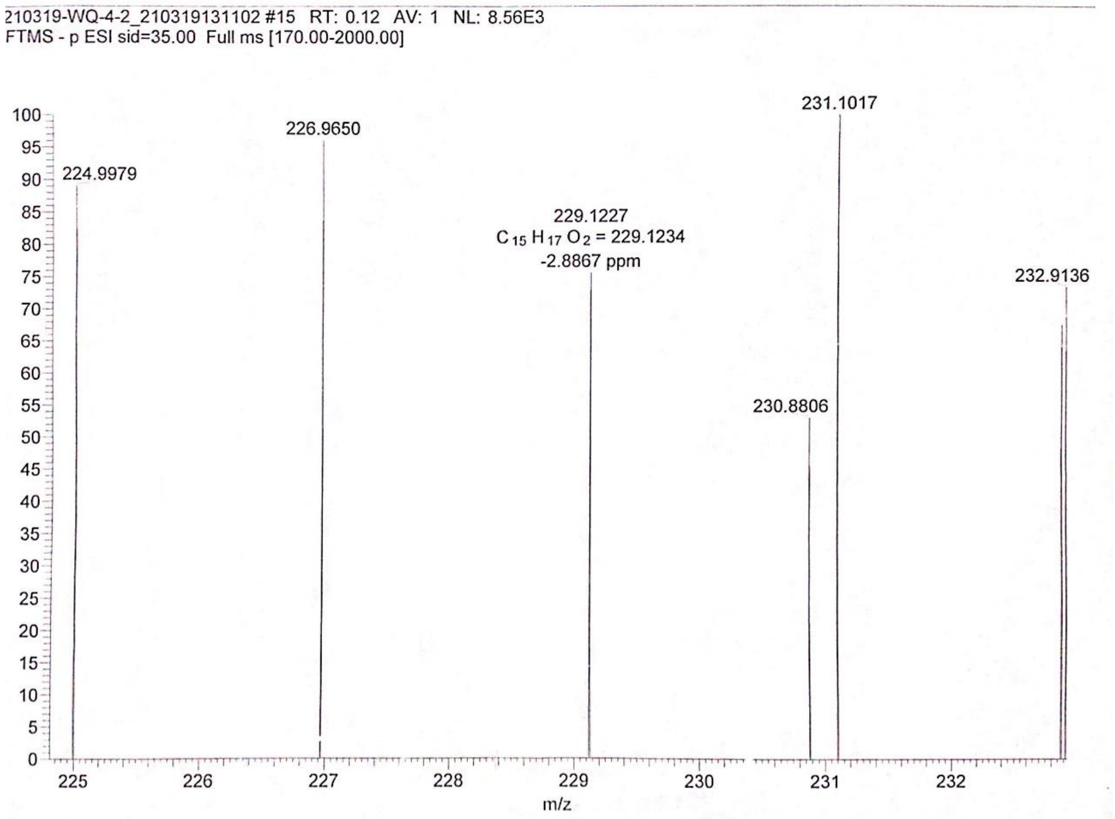

Figure S15. The HRESIMS spectrum of compound **2**.

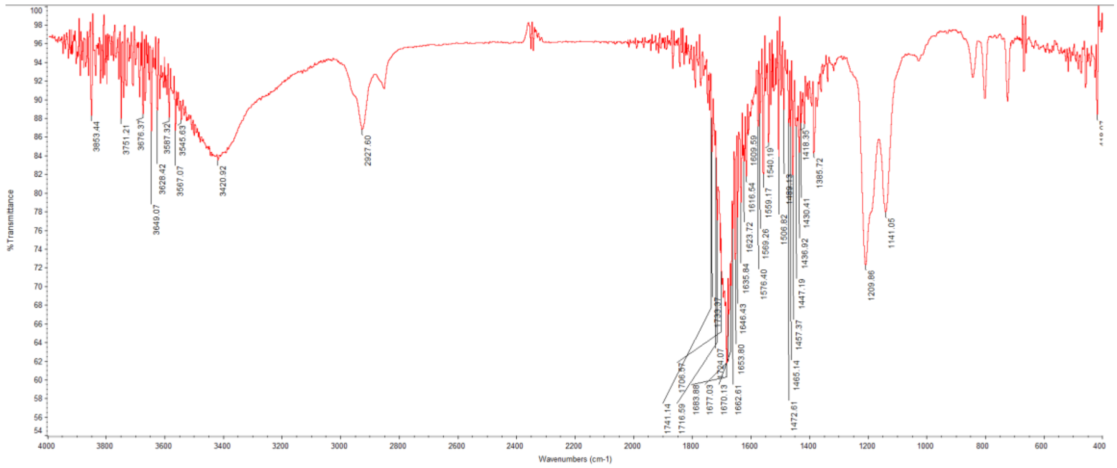

Figure S16. The IR spectrum of compound **2**.

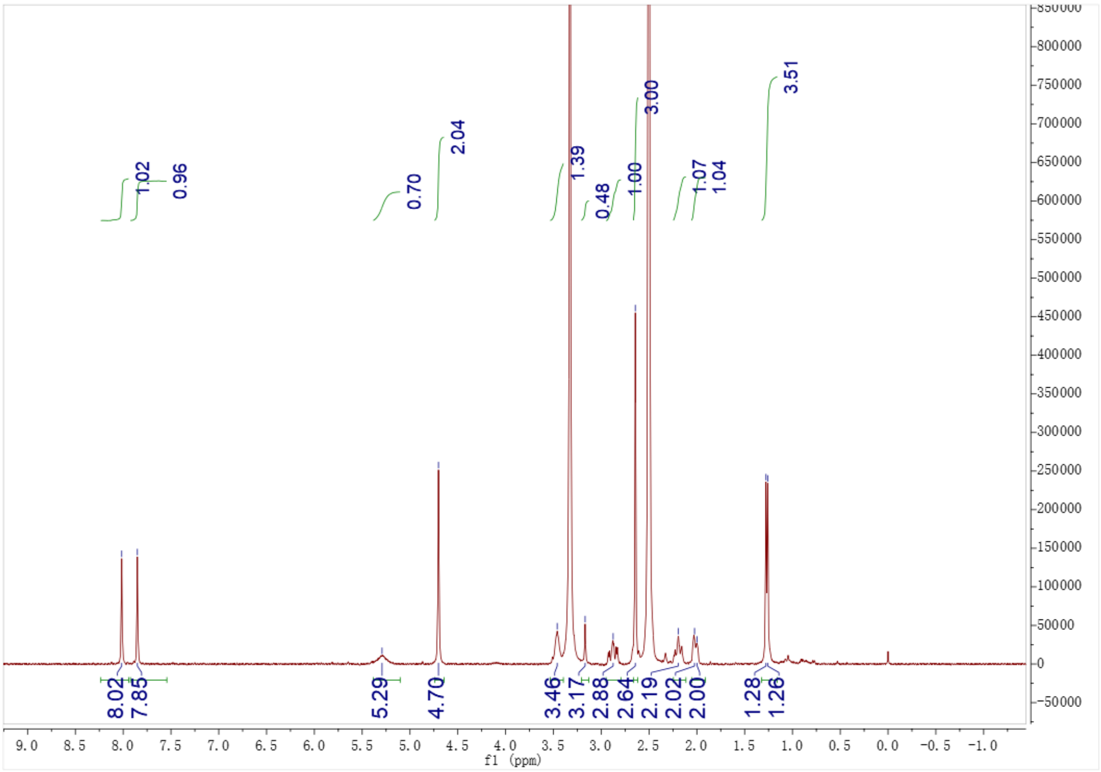

Figure S17. ^{1}H NMR (400 MHz, DMSO) spectrum of compound **3**.

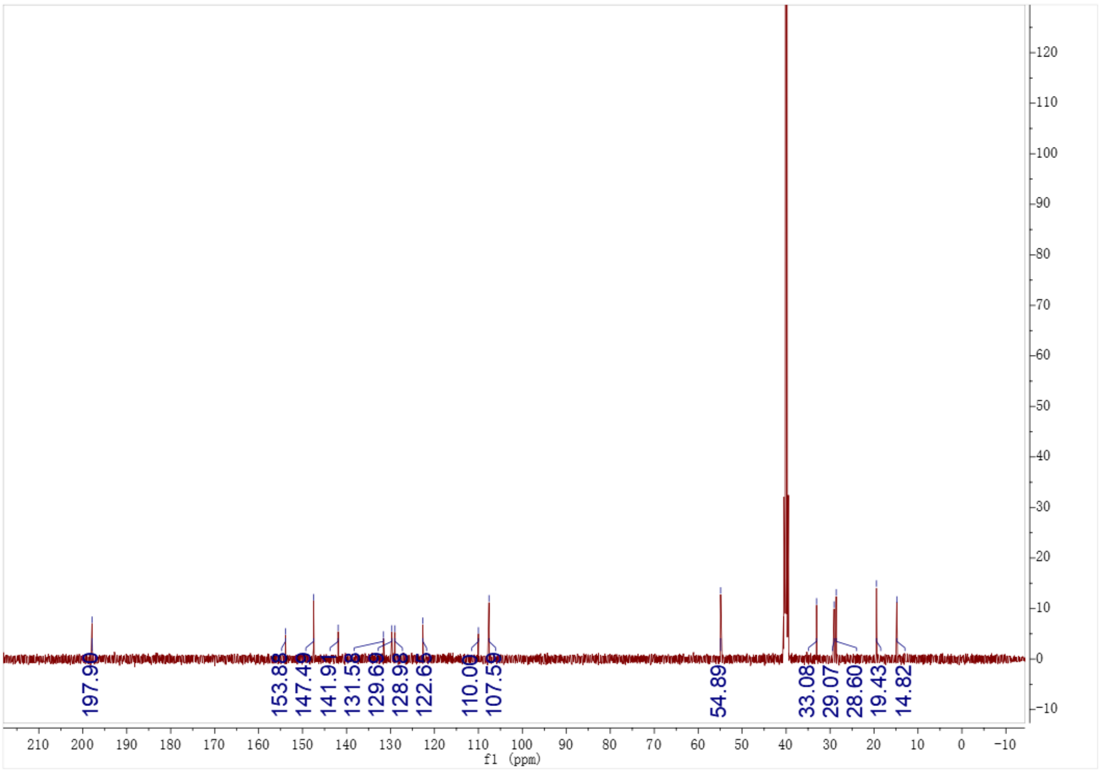

Figure S18. ^{13}C NMR (125 MHz, DMSO) spectrum of compound **3**.

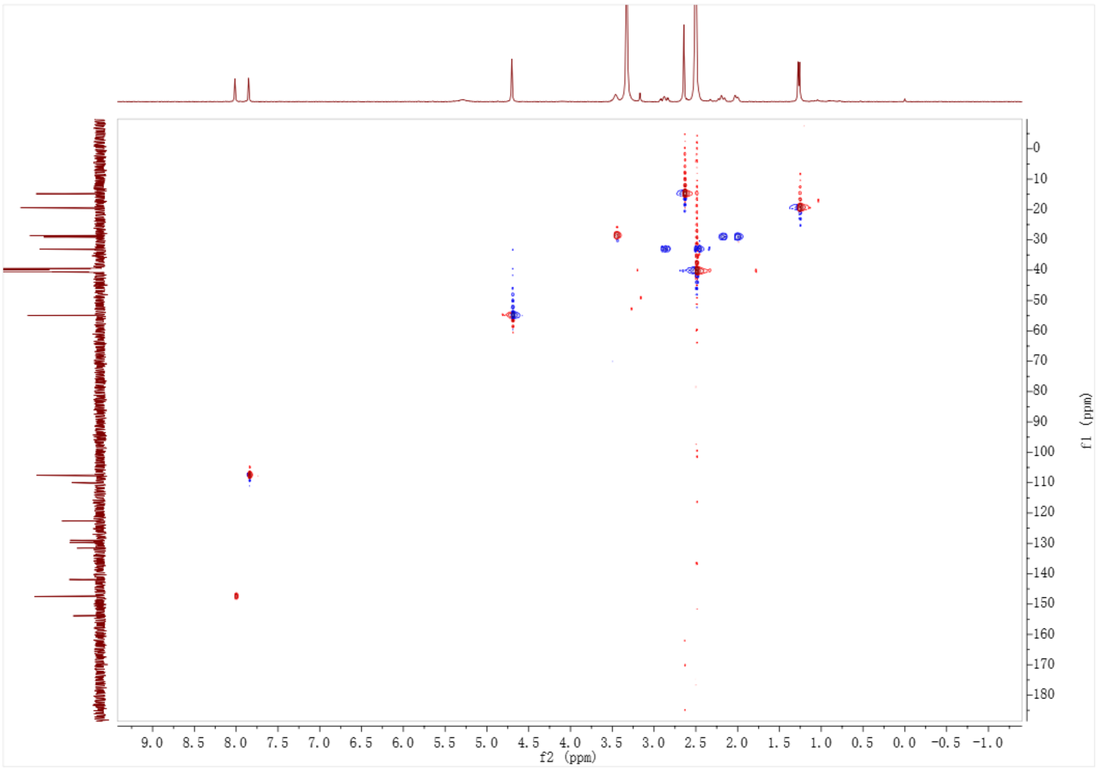

Figure S19. HSQC spectrum of compound **3**.

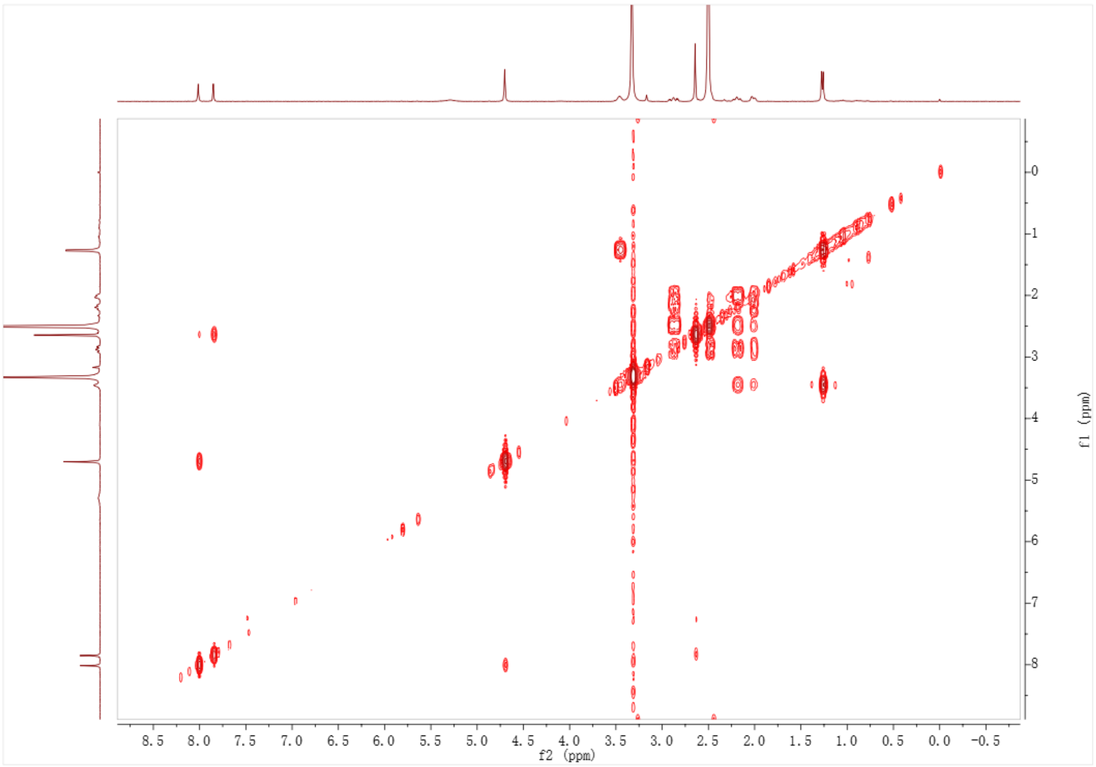

Figure S20. ^{1}H-^{1}H COSY (500 MHz, DMSO) spectrum of compound **3**.

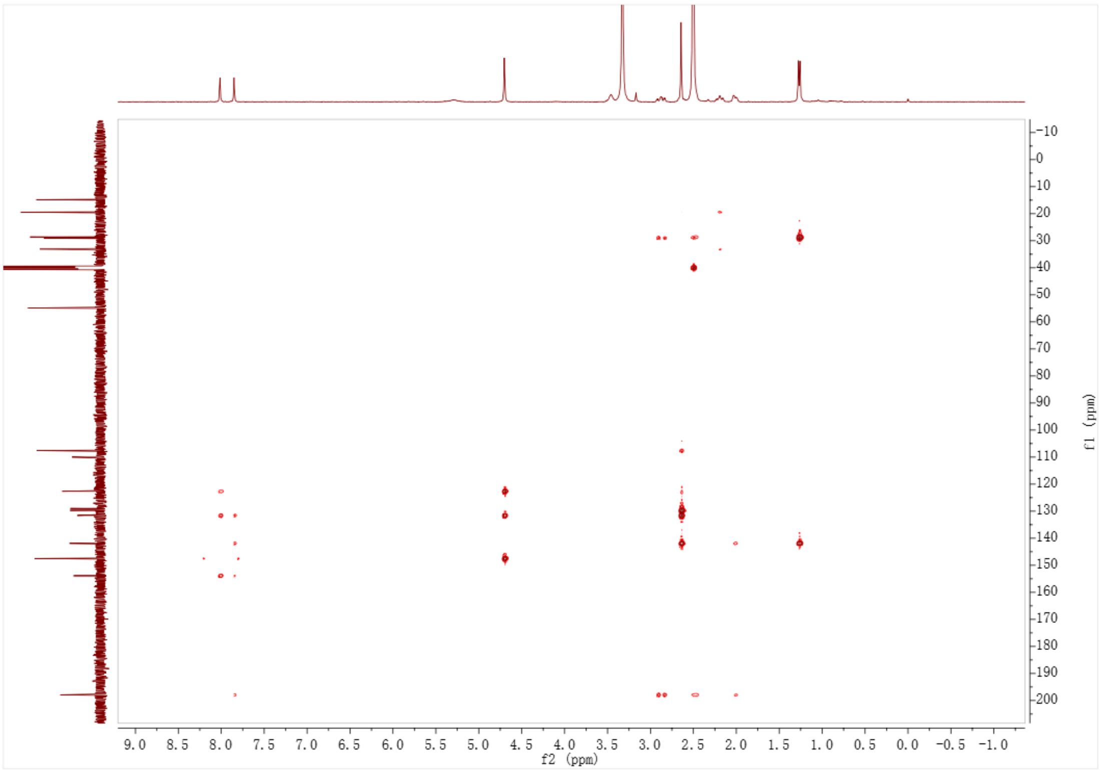

Figure S21. HMBC spectrum of compound **3**.

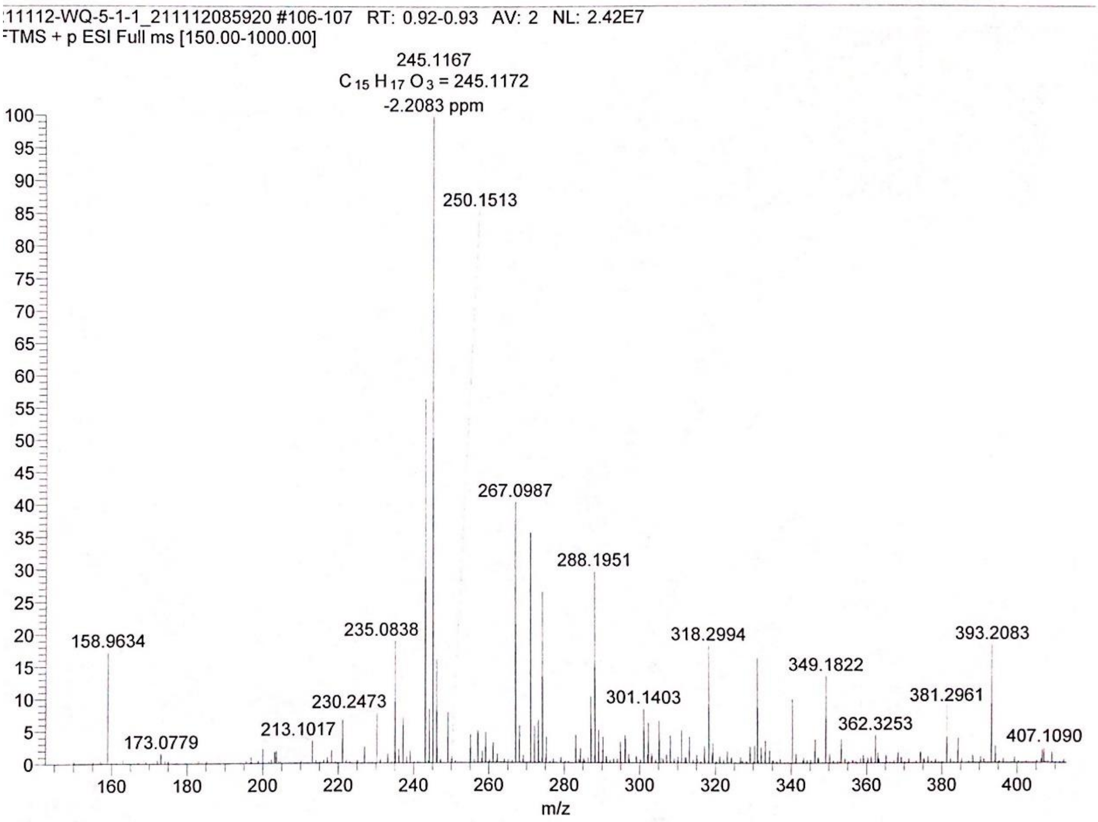

Figure S22. The HRESIMS spectrum of compound **3**.

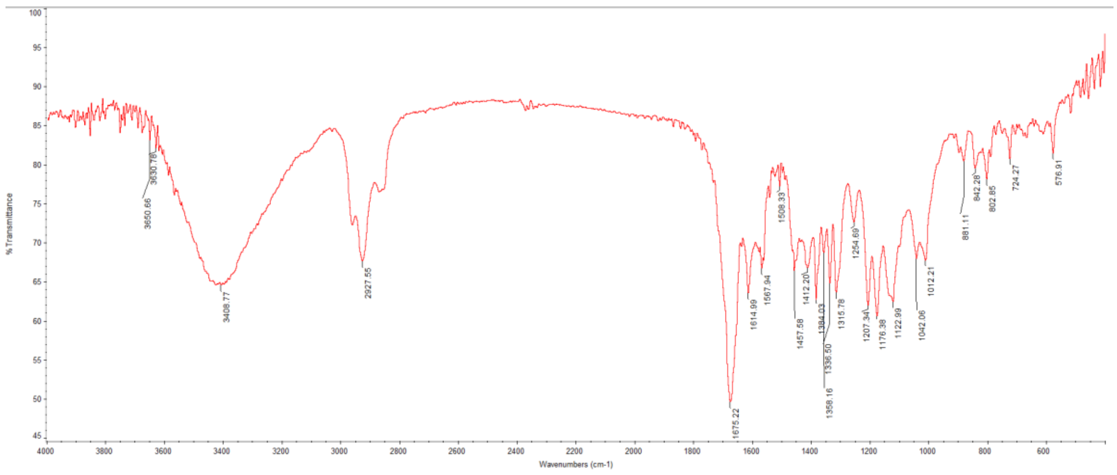

Figure S23. The IR spectrum of compound **3**.

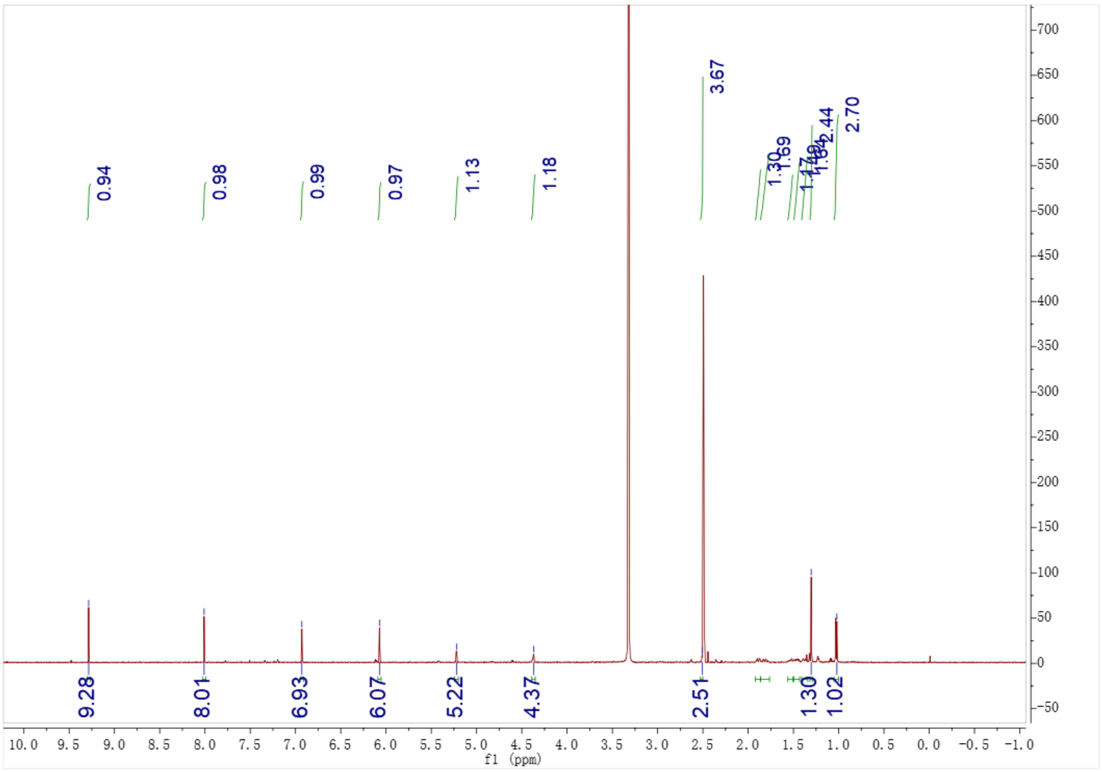

Figure S24. ^{1}H NMR (500 MHz, DMSO) spectrum of compound **4**.

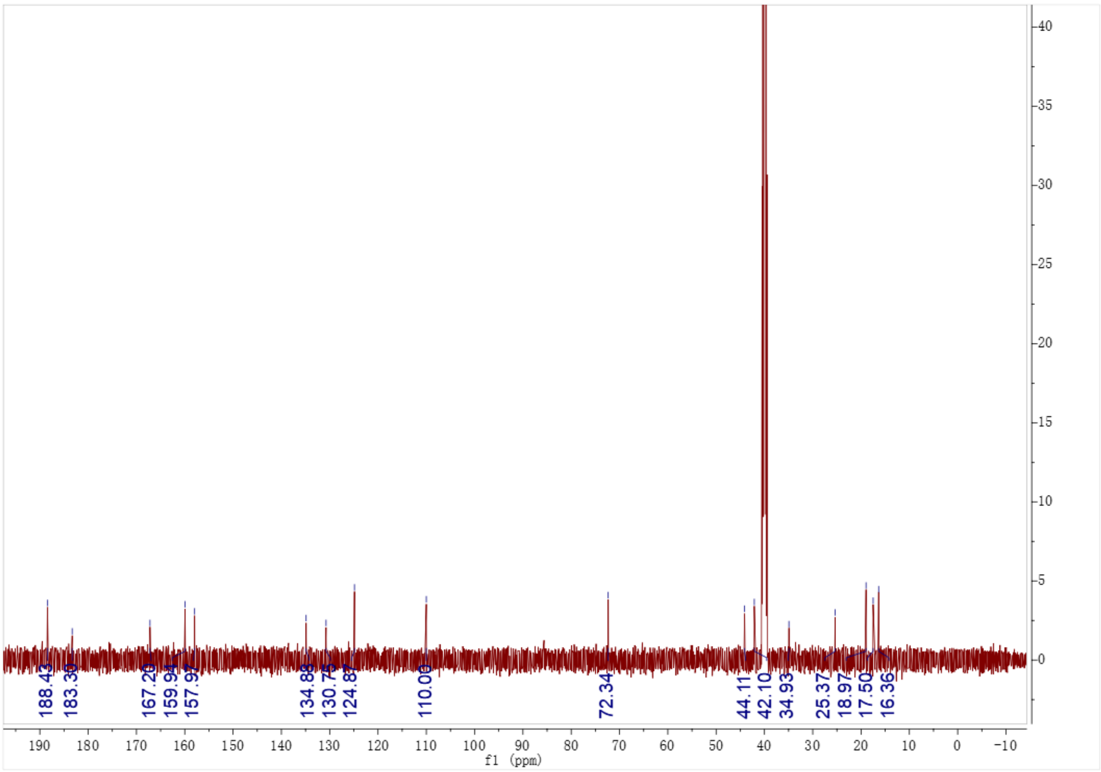

Figure S25. ^{13}C NMR (125 MHz, DMSO) spectrum of compound **4**.

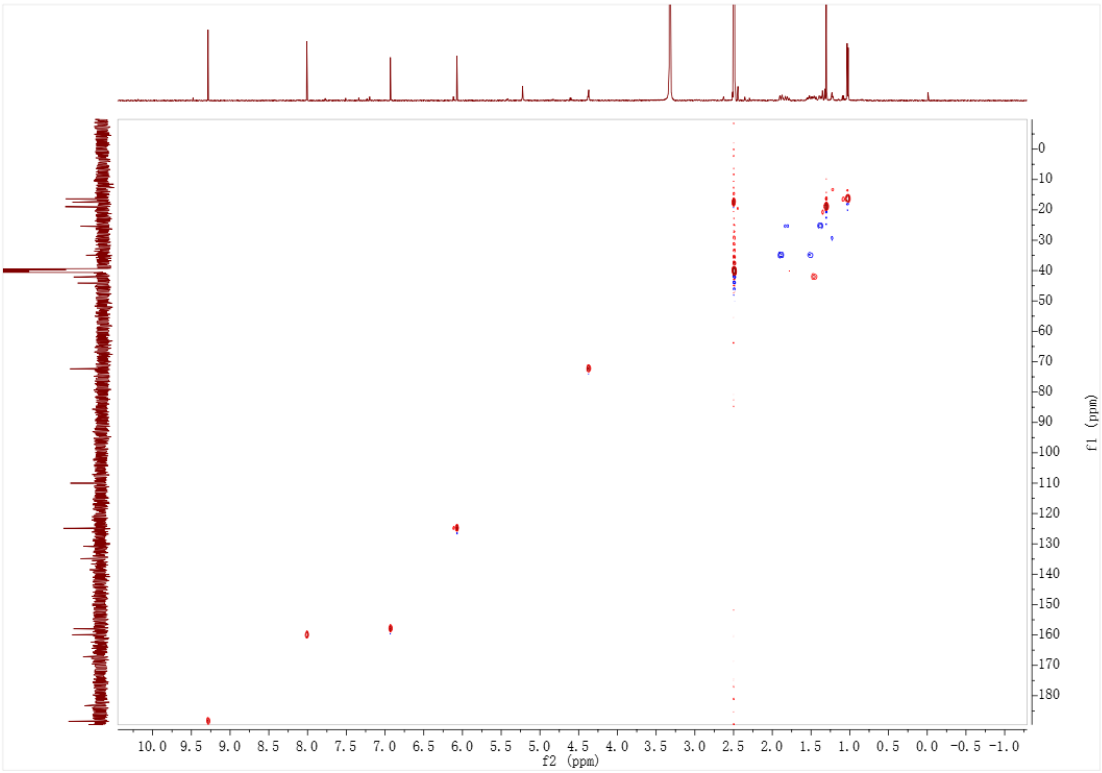

Figure S26. HSQC spectrum of compound **4**.

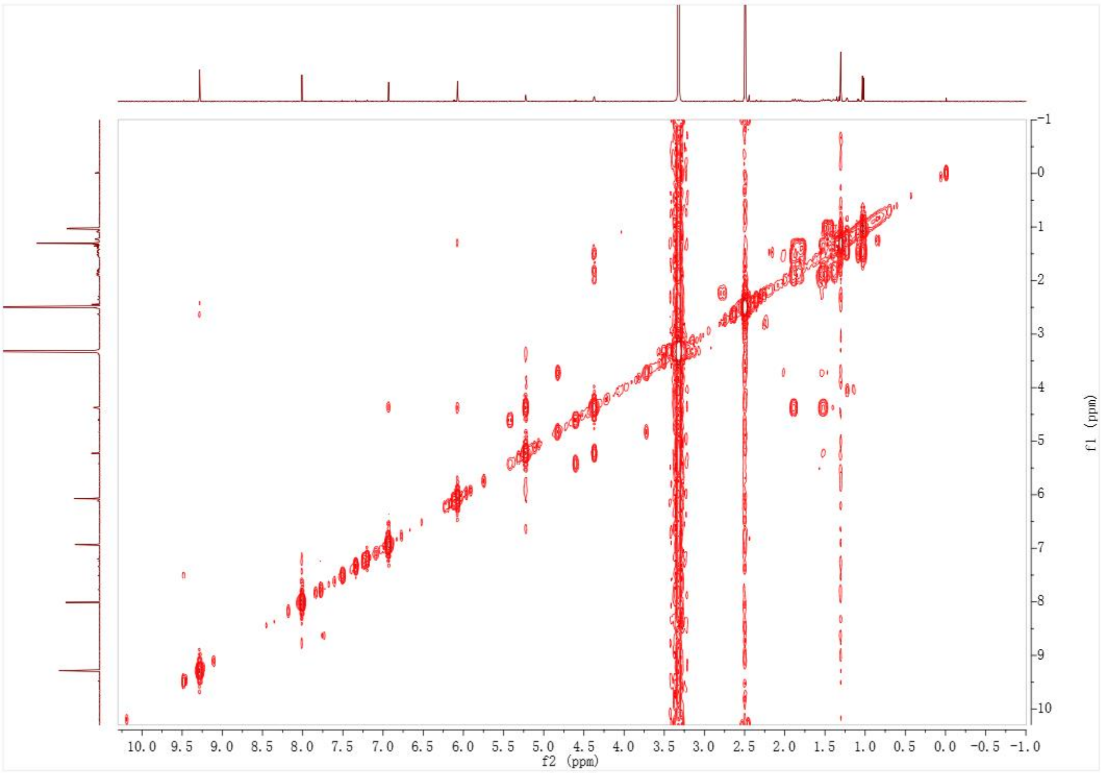

Figure S27. ^{1}H-^{1}H COSY (500 MHz, DMSO) spectrum of compound **4**.

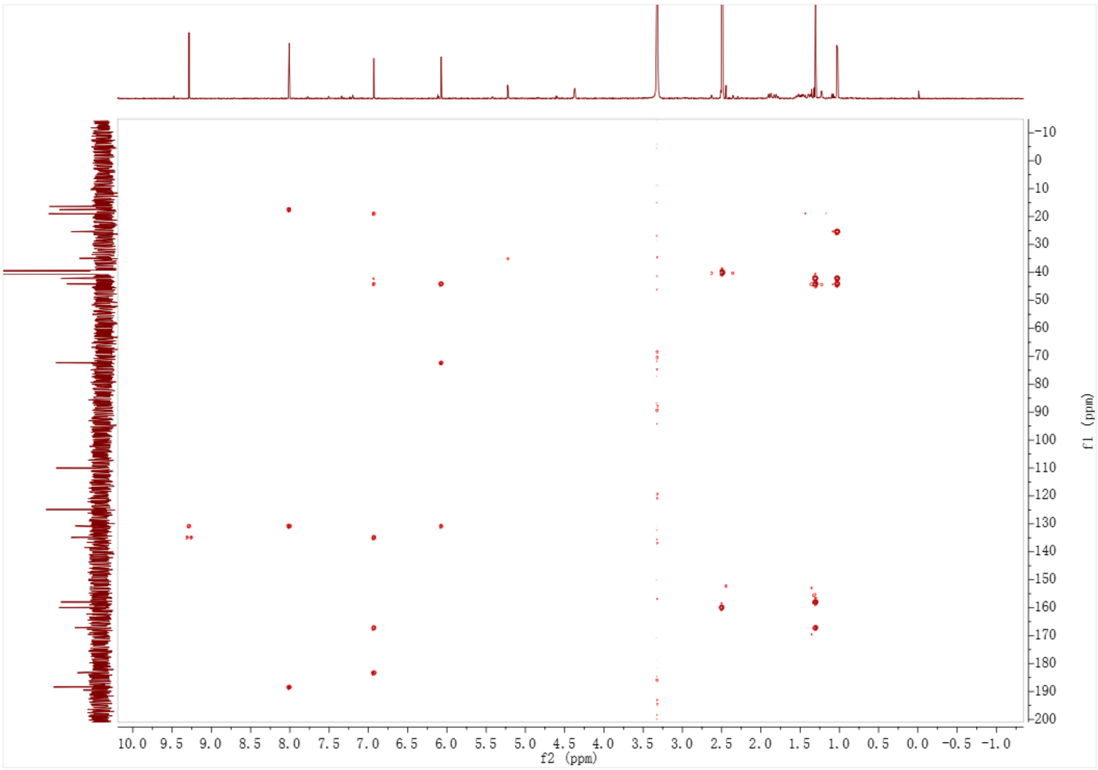

Figure S28. HMBC spectrum of compound **4**.

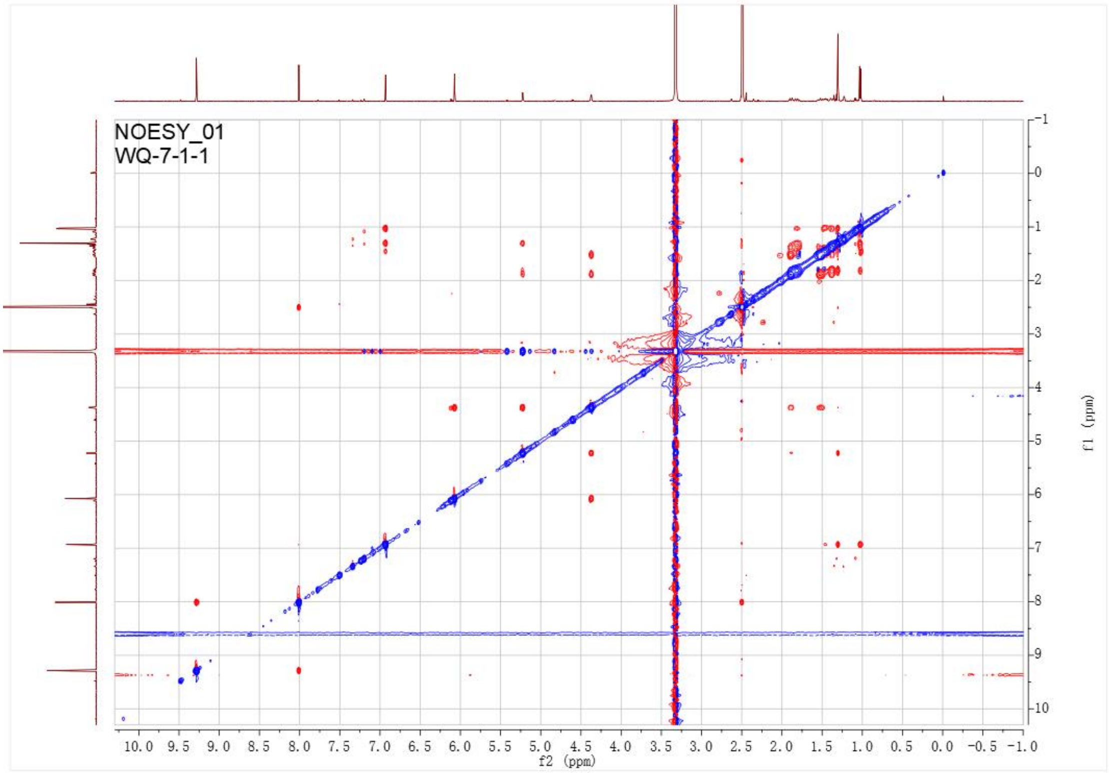

Figure S29. NOESY (500 MHz, DMSO) spectrum of compound **4**.

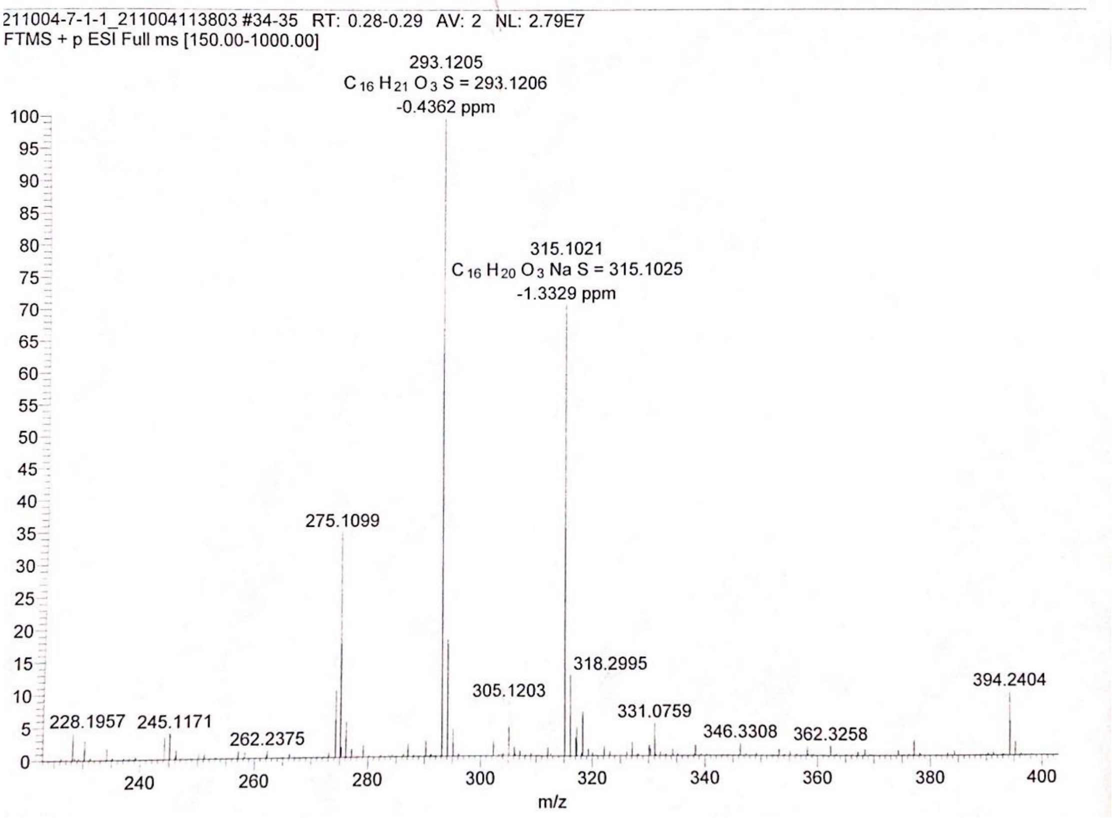

Figure S30. The HRESIMS spectrum of compound **4**.

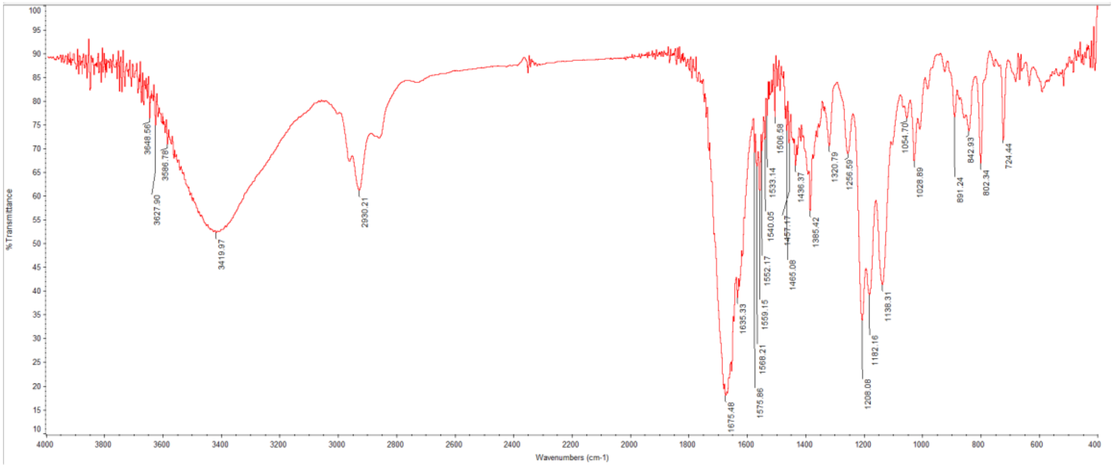

Figure S31. The IR spectrum of compound **4**.

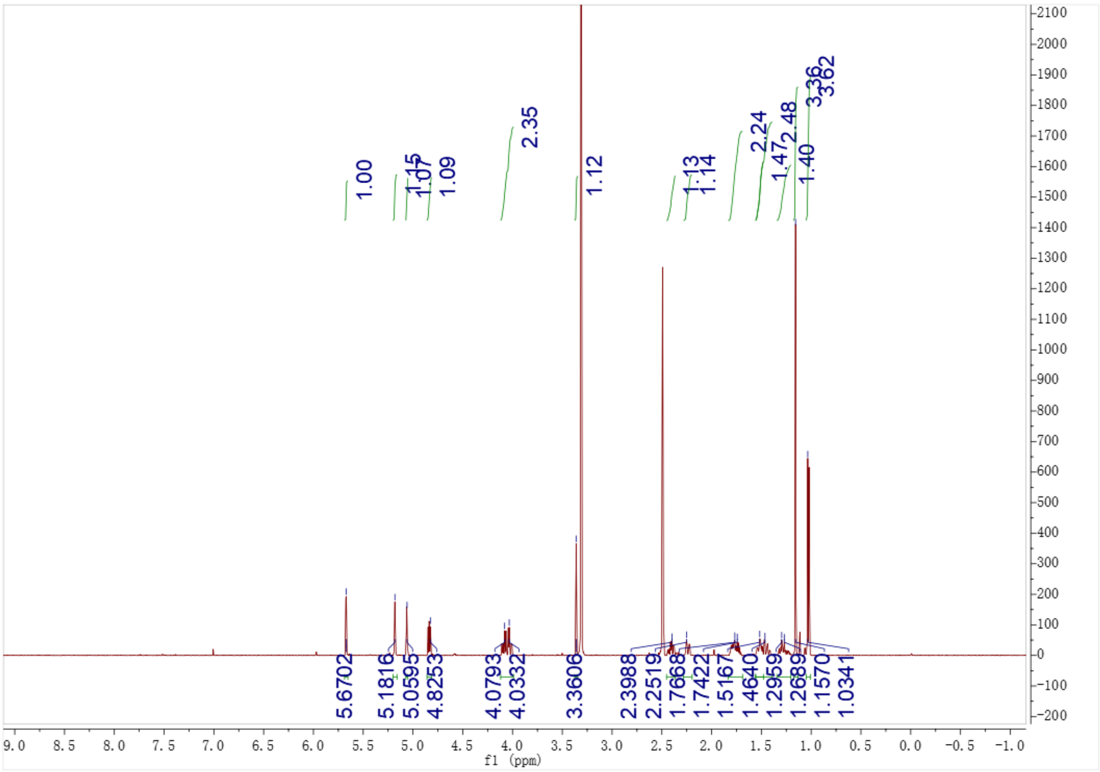

Figure S32. ^{1}H NMR (500 MHz, DMSO) spectrum of compound **5**.

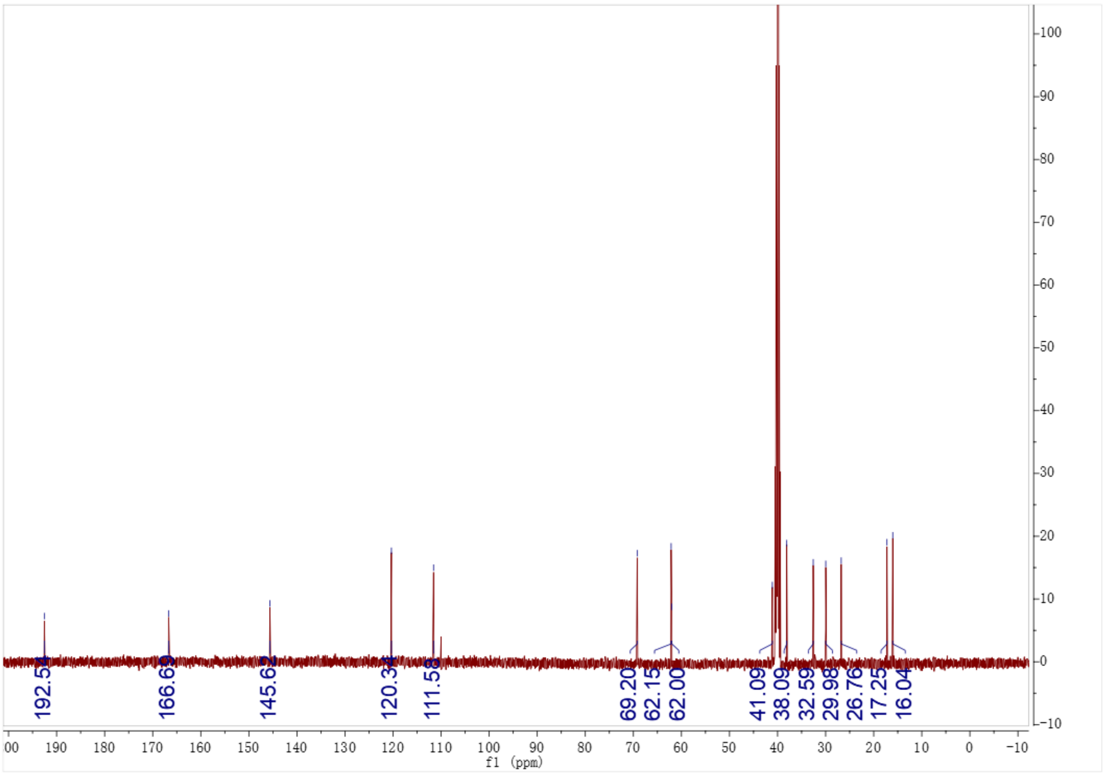

Figure S33. ^{13}C NMR (125 MHz, DMSO) spectrum of compound **5**.

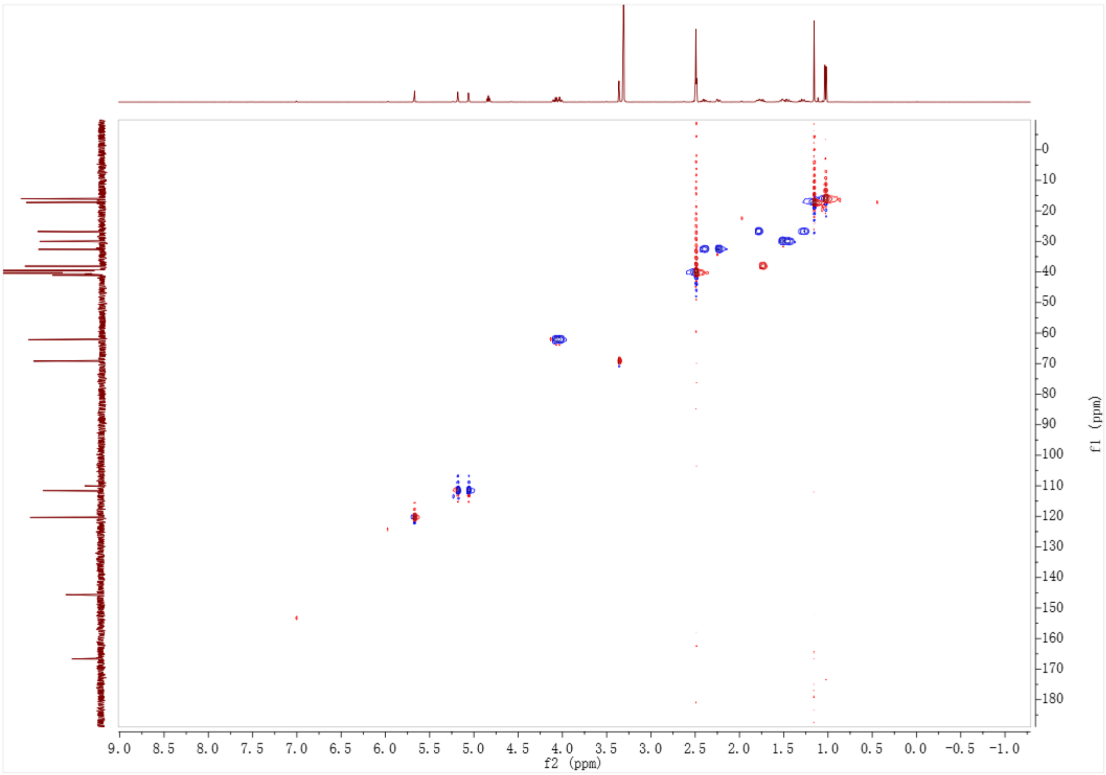

Figure S34. HSQC spectrum of compound **5**.

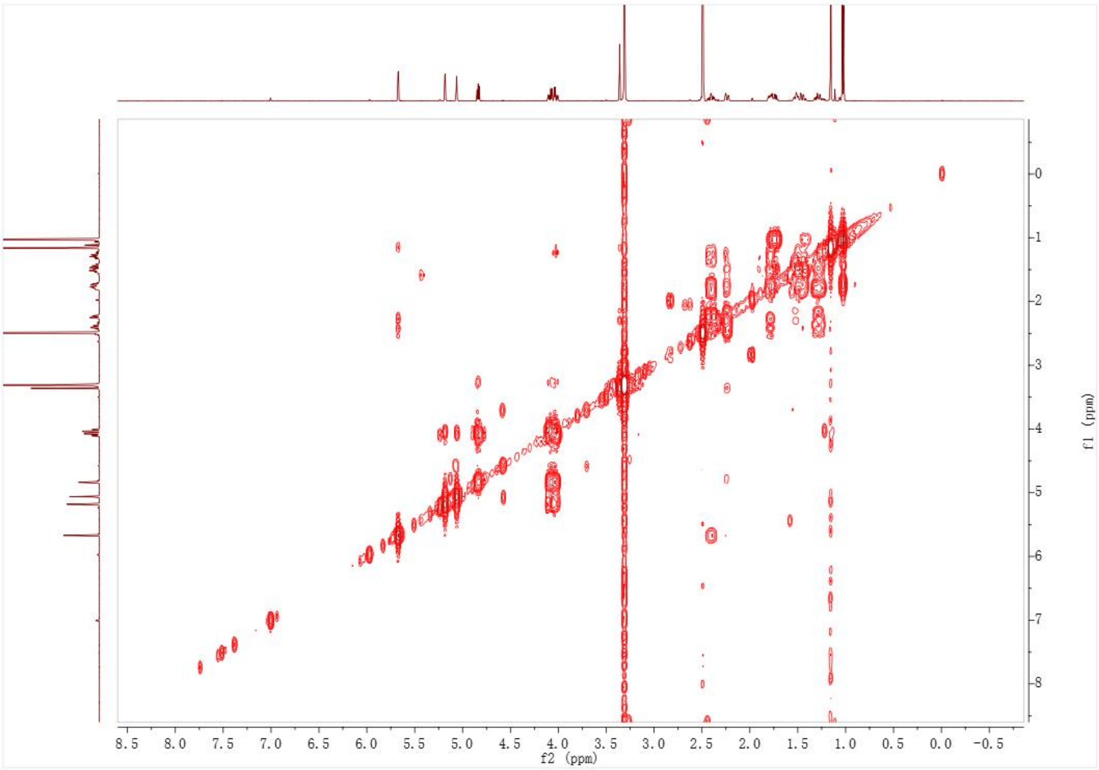

Figure S35. ^{1}H-^{1}H COSY (500 MHz, DMSO) spectrum of compound **5**.

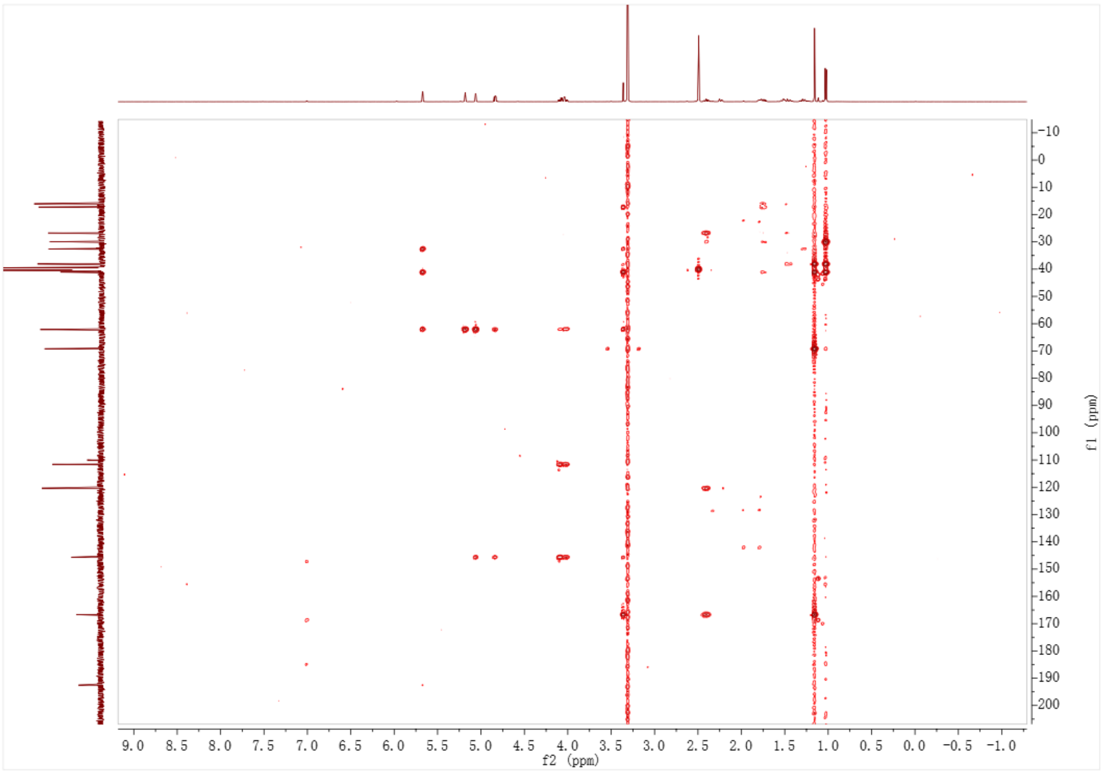

Figure S36. HMBC spectrum of compound **5**.

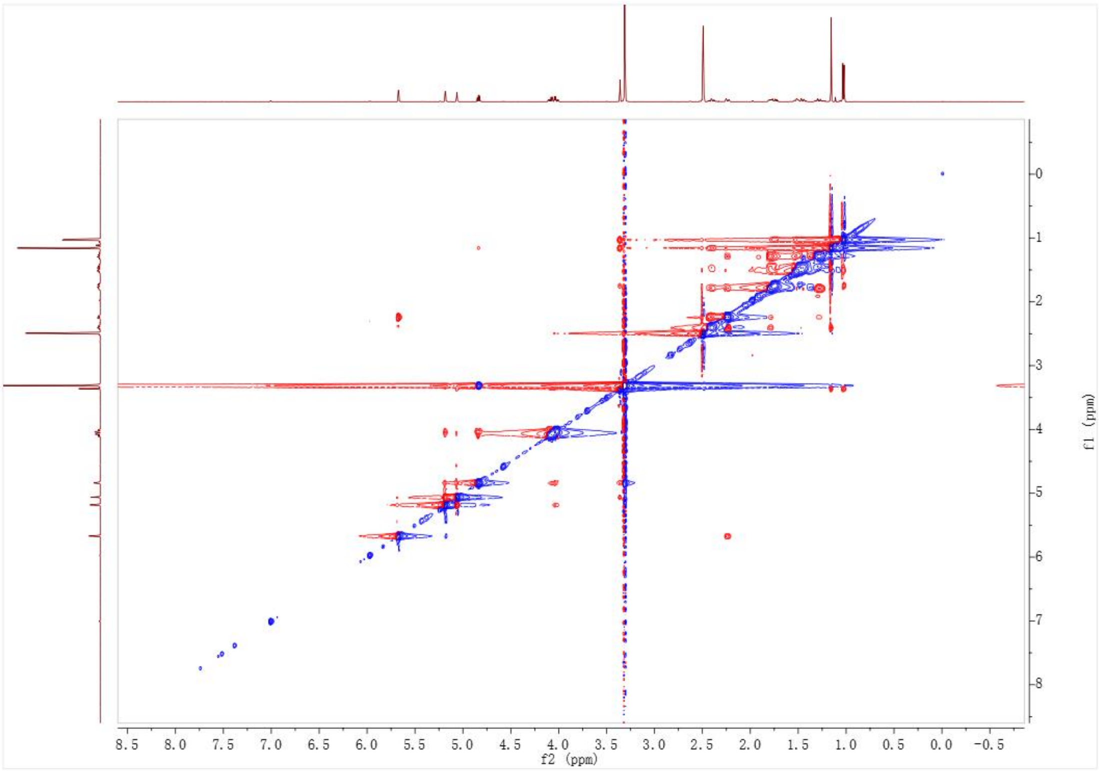

Figure S37. NOESY (500 MHz, DMSO) spectrum of compound **5**.

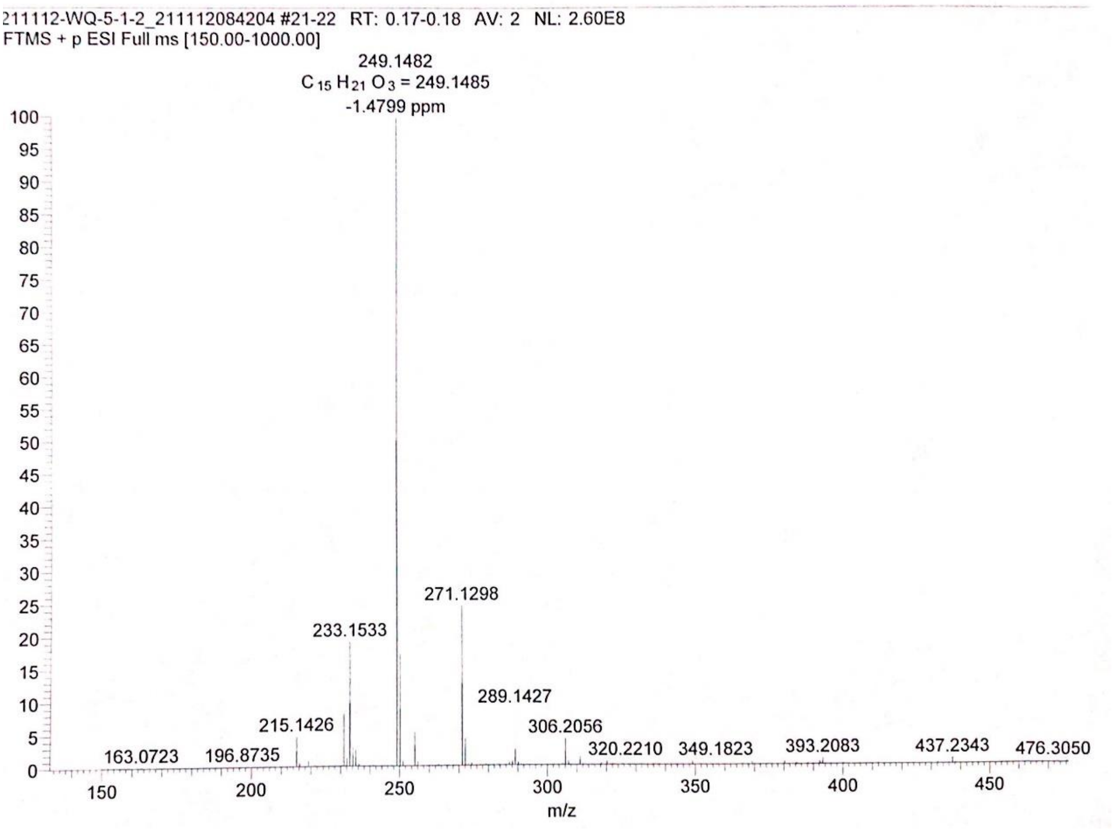

Figure S38. The HRESIMS spectrum of compound **5**.

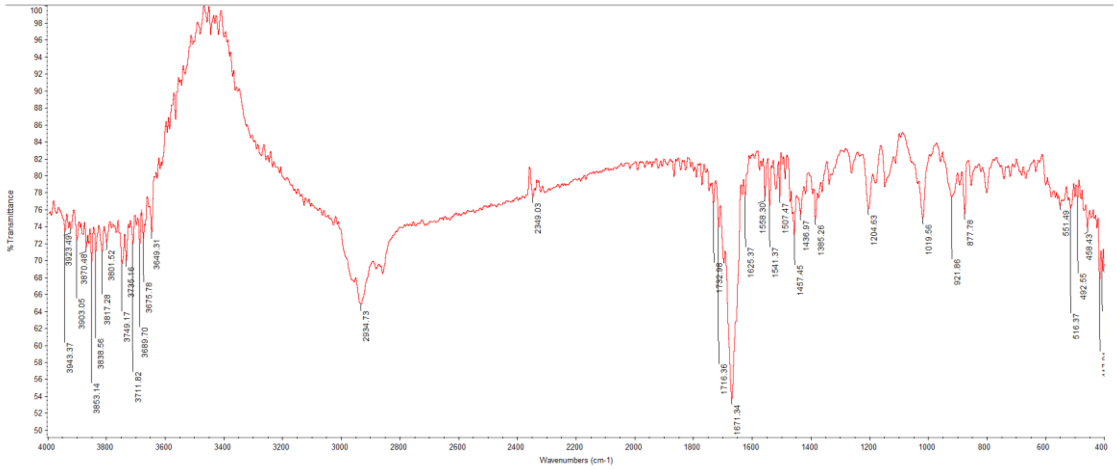

Figure S39. The IR spectrum of compound **5**.

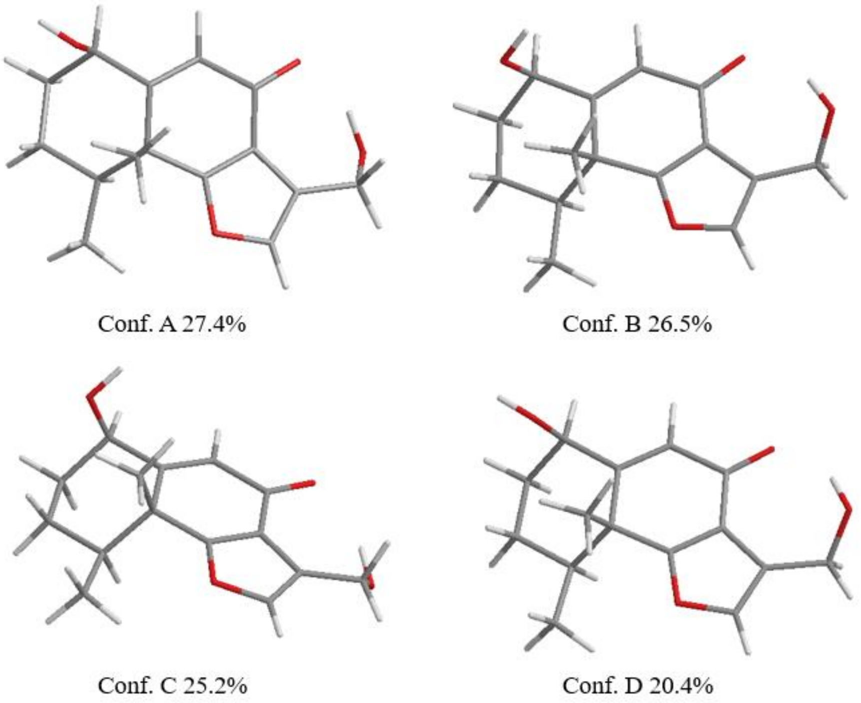

Figure S40. DFT-optimized structures for low-energy conformers of (1*R*, 4*S*, 5*R*)-**1** at B3LYP/TZVP level in DMSO (CPCM) (Conformer populations were calculated using the Gibbs free energy and Boltzmann population at 298 K estimated thereof).

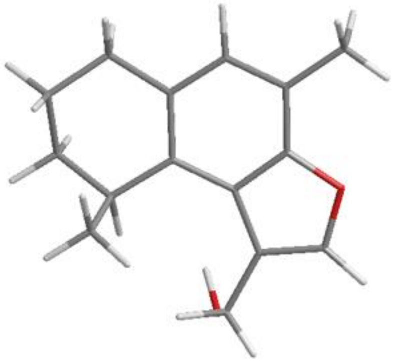

Conf. A 37.7%

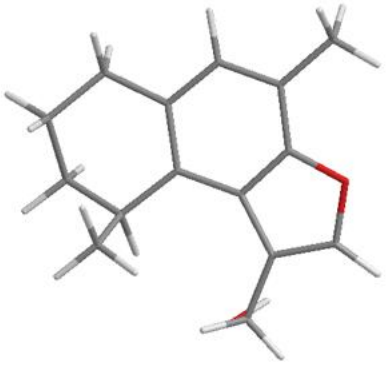

Conf. B 32.4%

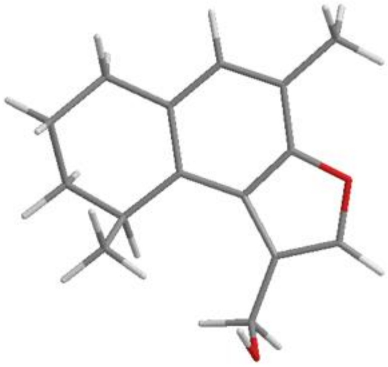

Conf. C 17.4%

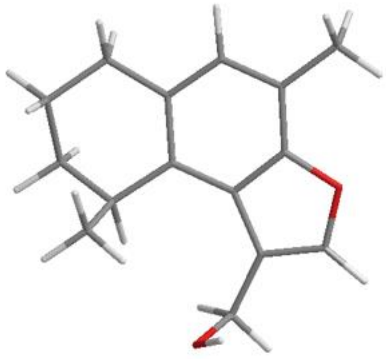

Conf. D 11.2%

Figure S41. DFT-optimized structures for low-energy conformers of (4*R*)-**2** at B3LYP/TZVP level in DMSO (CPCM) (Conformer populations were calculated using the Gibbs free energy and Boltzmann population at 298 K estimated thereof).

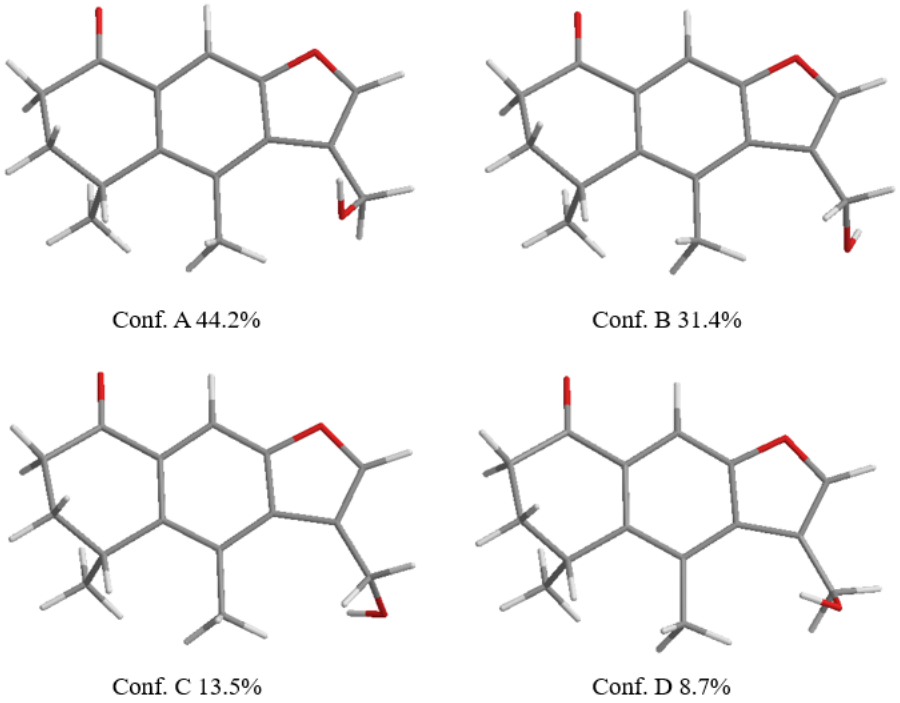

Figure S42. DFT-optimized structures for low-energy conformers of (4*R*)-**3** at B3LYP/TZVP level in DMSO (CPCM) (Conformer populations were calculated using the Gibbs free energy and Boltzmann population at 298 K estimated thereof).

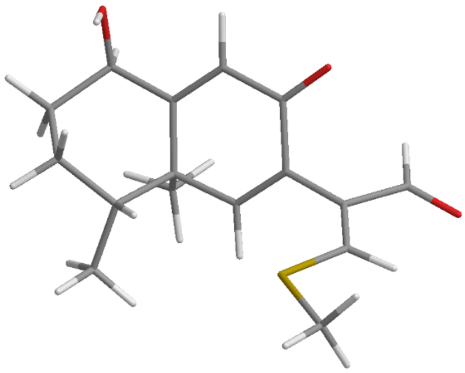

Conf. A 29.2%

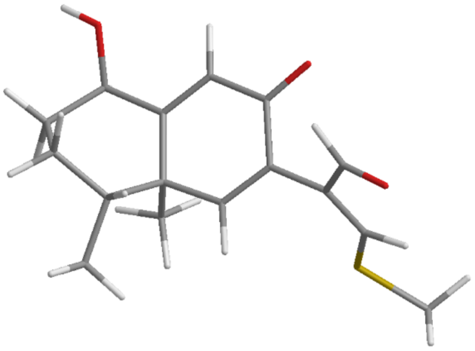

Conf. B 25.4%

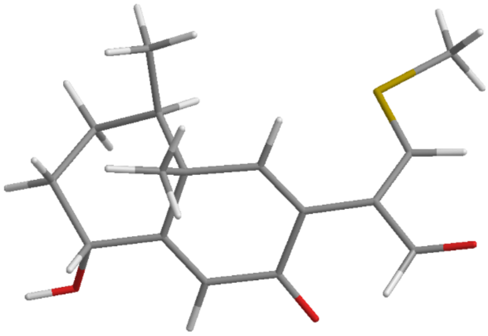

Conf. C 20.2%

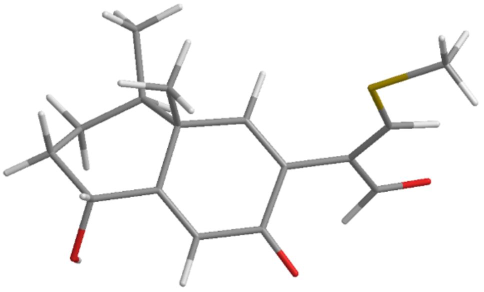

Conf. D 17.5%

Figure S43. DFT-optimized structures for low-energy conformers of (4*R*)-**4** at B3LYP/TZVP level in DMSO (CPCM) (Conformer populations were calculated using the Gibbs free energy and Boltzmann population at 298 K estimated thereof).

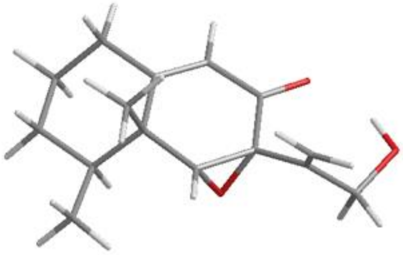

Conf. A 59.9%

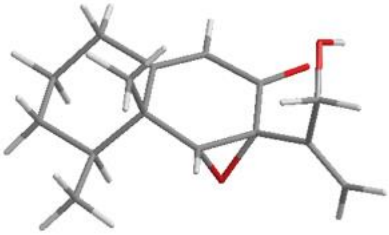

Conf. B 21.6%

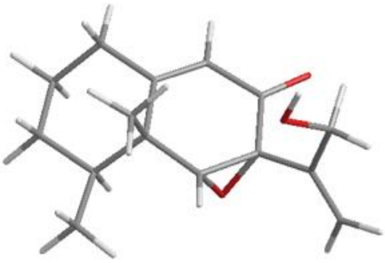

Conf. C 7.6%

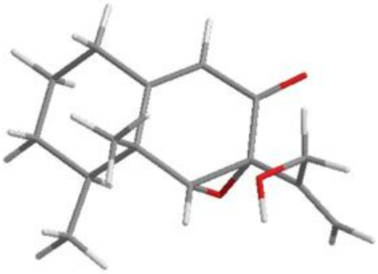

Conf. D 6.0%

Figure S44. DFT-optimized structures for low-energy conformers of (4*S*,5*R*,6*R*,7*R*)-**5**

at B3LYP/TZVP level in DMSO (CPCM) (Conformer populations were calculated using the Gibbs free energy and Boltzmann population at 298 K estimated thereof).

Conformations	Frequencies		
	1	2	3
A	46.2460	61.6322	93.1044
B	45.9745	61.4710	94.3548
C	49.0526	61.3063	91.5809
D	45.5606	62.5549	94.2921

Table S1. Harmonic frequencies (cm**-**1**) of **1**.

Conformations	Frequencies		
	1	2	3
A	67.1076	77.8603	93.8040
B	63.8522	79.6450	94.4259
C	56.1423	74.2237	87.9819
D	32.3779	71.3376	88.5621

Table S2. Harmonic frequencies (cm**-**2**) of **2**.

Conformations	Frequencies		
	1	2	3
A	50.2389	53.1863	73.1078
B	51.6273	55.2291	82.2034
C	39.4457	53.4979	73.1831
D	48.8863	56.5290	83.2251

Table S3. Harmonic frequencies (cm**-**3**) of **3**.

Conformations	Frequencies		
	1	2	3
A	24.1409	37.0744	54.0982

B	20.6730	40.0519	57.5593
C	25.1176	37.2193	54.8739
D	21.1952	40.7051	57.6114

Table S4. Harmonic frequencies (cm**-**4**) of **4**.

Conformations	Frequencies		
	1	2	3
A	39.6984	54.1896	83.1232
B	40.4606	43.0203	82.7451
C	33.1236	54.1258	60.0852
D	28.4078	52.0435	60.7965

Table S5. Harmonic frequencies (cm**-**5**) of **5**.

Conformations	E+ZPE	G	%
A	-883.034519	-883.077920	27.4
B	-883.035662	-883.078906	26.5
C	-883.035786	-883.079093	25.2
D	-883.034512	-883.077911	20.4

Table S6. Important thermodynamic parameters (a.u.) and Boltzmann distributions of the optimized-1 at B3LYP/TZVP level of theory with PCM solvent model for DMSO. E+ZPE, G: total energy with zero-point energy (ZPE) and Gibbs free energy at B3LYP/TZVP level of theory with PCM solvent model for DMSO. %: Boltzmann distributions, using the relative Gibbs free energies as weighting factors

Conformations	E+ZPE	G	%
A	-732.614420	-732.656399	37.7
B	-732.616380	-732.658410	32.4
C	-732.612141	-732.654485	17.4
D	-732.613909	-732.656465	11.2

Table S7. Important thermodynamic parameters (a.u.) and Boltzmann distributions of the optimized-2 at B3LYP/TZVP level of theory with PCM solvent model for DMSO.

Conformations	E+ZPE	G	%
A	-806.660788	-806.703905	44.2
B	-806.660883	-806.703900	31.4
C	-806.658457	-806.701935	13.5

D	-806.658376	-806.701177	8.7

Table S8. Important thermodynamic parameters (a.u.) and Boltzmann distributions of the optimized-**3** at B3LYP/TZVP level of theory with PCM solvent model for DMSO.

Conformations	E+ZPE	G	%
A	-1245.272181	-1245.322236	29.2
B	-1245.272372	-1245.322440	25.4
C	-1245.272161	-1245.322083	20.2
D	-1245.272195	-1245.322129	17.5

Table S9. Important thermodynamic parameters (a.u.) and Boltzmann distributions of the optimized-4 at B3LYP/TZVP level of theory with PCM solvent model for DMSO.

Conformations	E+ZPE	G	%
A	-808.959176	-809.002888	59.9
B	-808.957473	-809.001675	21.6
C	-808.957153	-809.001814	7.6
D	-808.957633	-809.002222	6.0

Table S10. Important thermodynamic parameters (a.u.) and Boltzmann distributions of the optimized-5 at B3LYP/TZVP level of theory with PCM solvent model for DMSO.
